# The biophysical property of the limbal niche maintains stemness through YAP

**DOI:** 10.1038/s41418-023-01156-7

**Published:** 2023-04-24

**Authors:** Swarnabh Bhattacharya, Abhishek Mukherjee, Sabrina Pisano, Shalini Dimri, Eman Knaane, Anna Altshuler, Waseem Nasser, Sunanda Dey, Lidan Shi, Ido Mizrahi, Noam Blum, Ophir Jokel, Aya Amitai-Lange, Anna Kaganovsky, Michael Mimouni, Sergiu Socea, Mohamad Midlij, Beatrice Tiosano, Peleg Hasson, Chloe Feral, Haguy Wolfenson, Ruby Shalom-Feuerstein

**Affiliations:** 1 Department of Genetics & Developmental Biology, The Rappaport Faculty of Medicine & Research Institute, Technion Integrated Cancer Center, Technion - Israel Institute of Technology, 31096 Haifa, Israel; 2 Department of Medical Oncology and Center for Functional Cancer Epigenetics, Dana–Farber Cancer Institute, Boston, MA 02215 USA; 3 Departments of Medicine, Brigham & Women’s Hospital and Harvard Medical School, Boston, MA 02115 USA; 4 Université Côte d’Azur, INSERM, CNRS, IRCAN, 06107 Nice, France; 5 Department of Ophthalmology, Rambam Health Care Campus, 31096 Haifa, Israel; 6 Department of Ophthalmology, Hilel Yafe Medical Center, Hadera, Israel

**Keywords:** Gene expression, Cancer stem cells, Gene regulation

## Abstract

The cell fate decisions of stem cells (SCs) largely depend on signals from their microenvironment (niche). However, very little is known about how biochemical niche cues control cell behavior in vivo. To address this question, we focused on the corneal epithelial SC model in which the SC niche, known as the limbus, is spatially segregated from the differentiation compartment. We report that the unique biomechanical property of the limbus supports the nuclear localization and function of Yes-associated protein (YAP), a putative mediator of the mechanotransduction pathway. Perturbation of tissue stiffness or YAP activity affects SC function as well as tissue integrity under homeostasis and significantly inhibited the regeneration of the SC population following SC depletion. In vitro experiments revealed that substrates with the rigidity of the corneal differentiation compartment inhibit nuclear YAP localization and induce differentiation, a mechanism that is mediated by the TGFβ−SMAD2/3 pathway. Taken together, these results indicate that SC sense biomechanical niche signals and that manipulation of mechano-sensory machinery or its downstream biochemical output may bear fruits in SC expansion for regenerative therapy.

## Introduction

Stem cell (SC) fate decisions are controlled by extrinsic signals derived from the local microenvironment, known as the niche [1, 2]. When departing away from the niche, SCs sense the lack of self-renewal signals or the presence of commitment cues and respond by activating differentiation programs. Likewise, following SC loss, committed cells repopulate the niche and undergo reprogramming into bona fide SCs, a process that depends on an intact niche [3–6]. To date, however, very little is known about SC–niche crosstalk in vivo. A better understanding of SC regulation by the niche is important to clarify mechanisms of SC function under homeostasis, involvement in aging, and pathology. Current SC cultures are very limited by the gap in knowledge of niche essential factors. Consequently, primary cultures typically sustain for very short periods, as SCs seemingly lose self-renewal and long-term proliferation capacity when grown outside the niche in the culture dish [7, 8]. This deficit severely halts SC application in regenerative medicine.

SC responses to mechanical cues from various niche components are largely unknown and in recent years starting to be addressed in vivo [9]. Few adult SC niches seem to be softer than the differentiation compartments [10–13]. It is hypothesized that SCs can sense the biomechanical properties of their microenvironment and translate this signal into a decision to self-renew or differentiate. A key regulatory step in responses to mechanotransduction pathways involves changes in the localization of the co-transcriptional regulator, YAP, a process that seems to depend on tissue and cell type. In many cases, in response to stiff matrix sensing, the co-transcriptional regulator, YAP, translocates from the cytoplasm to the nucleus and activates mechano-responsive pathways [14, 15]. However, in a few recent studies, mouse mammary gland stromal fibroblasts [16], murine incisor SCs [17], and muscle SCs [18] show opposite responses of YAP localization to matrix stiffness or mechanotransduction stimulation by the Rho-A pathway. A better understanding of YAP upstream regulators is needed to illuminate the differential impact on YAP in specific cell types, in response to substrate rigidity.

The corneal epithelium is excellent in vivo model to study the SC niche. The SCs reside in a well-demarcated domain of the limbus, at the ring-shaped zone of the tissue boundary with neighboring conjunctiva, whereas differentiated cells reside in the central cornea that is macroscopically distinguishable. Quiescent and active limbal SC populations function to constantly renew the murine corneal epithelium under homeostasis and efficiently repair central corneal injury [19–23]. Interestingly, the biomechanical rigidity of the human limbal niche is lower compared to that of the differentiated zone, and substrate stiffness was shown to induce limbal SC (LSC) differentiation [10, 13]. Contrasting studies report relatively higher [13, 24, 25] or lower [26] nuclear YAP levels in corneal differentiated cell compartment. Therefore, the influence of corneal stiffness on the subcellular localization of YAP remains unclear and its role in regulating LSC function under homeostasis, and in reprogramming of differentiated cells [5] is unknown.

Here we report that lower matrix rigidity of the limbus supports YAP activity to regulate the proliferation and wound healing response of LSCs, and the plasticity of committed cells. We propose that the higher corneal rigidity increases actomyosin contractility and renders YAP in the cytoplasm through a mechanism that is mediated by the activation of SMAD2/3. This mechanism might explain the cell type specific influence of matrix rigidity on the subcellular localization of YAP. Inhibiting the process of rigidity sensation or TGF-β pathway significantly enhances SC hallmarks and therefore should be considered when designing tissue engineering and SC-based therapies.

## Results

### YAP is essential for the maintenance of undifferentiated human LSC state

**Fig. 1:**
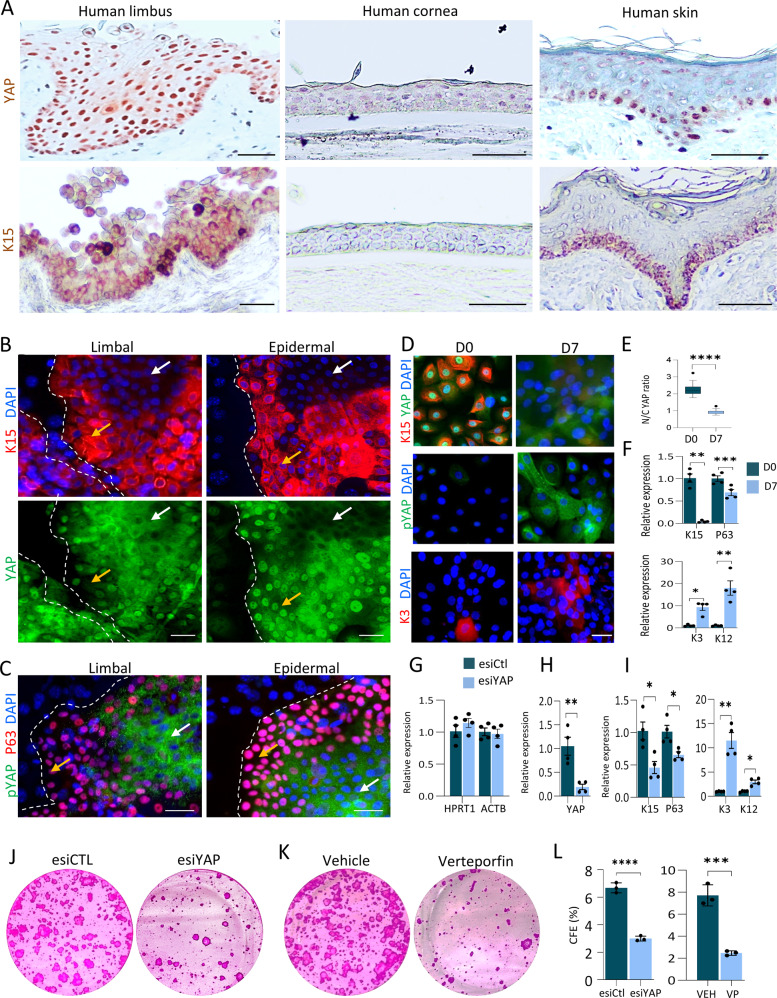
YAP is required to maintain LSC at undifferentiated state in vitro. **A** Immunohistochemistry of YAP and K15 was performed on paraffin sections human limbus, cornea, and skin. **B**,**C** Primary human limbal epithelial and foreskin epidermal cells were co-cultured with NIH-3T3-J2 feeder cells, grown on a plastic plate for 4 days and the expression of the indicated markers was examined by immunostaining. The golden and white arrows indicate stem/progenitor or differentiated cell regions, respectively. **D**–**F** Primary human LSCs were grown at low calcium (Day 0) or induced to differentiate in high calcium (Day 7) and the expression of the indicated markers was tested by immunostaining (**D**) that was further quantified for mean nuclear-to-cytoplasmic YAP intensity ratio (**E**) or by quantitative real-time PCR (qPCR) (**F**). **G**–**I** Primary human limbal epithelial cells were transfected with esiRNA against YAP (esiYAP) or control esiRNA (esiCtl) and 48–72 h later, the expression of housekeeping genes (**G**), YAP (**H**) and the indicated markers (**I**) was examined by qPCR. **J**–**L** Primary limbal epithelial cells were transfected with esiYAP or esiCtl or treated with Verteporfin or vehicle, and 48–72 h later, cells were subjected to a clonogenicity test (**J**, **K**) and the number of the colony was quantified (*n* = 3) (**L**). The qPCR data were normalized to the housekeeping gene *GAPDH* (mean ± standard error of mean, *n* = 4 biological replicates) as a fold increase compared to the control sample and the mean nuclear-to-cytoplasmic YAP intensity ratio from three independent experiments (**E**) is shown by the Tukey box-and-whisker plot followed by *t*-test with Welch’s correction. Statistical significance was assessed by *t*-test (*, *p* < 0.05; **, *p* < 0.01; ***, *p* < 0.001, ****, *p* < 0.0001). Immunostaining and Immunohistochemistry data are representative from 3 to 5 biological replicates. Nuclei were detected by DAPI counterstaining. Scale bars are 100 µm (**A**) and the rest are 50 µm.

To characterize YAP’s expression pattern, we first performed immunohistochemistry of YAP and K15 in human corneal tissue sections. Nuclear YAP (nYAP) was predominantly detected in the limbal compartment where K15^+^K12^−^ basal LSC reside (Figs. 1A, S1A). In addition, nYAP was occasionally detected in K15^+^K12^−^ limbal suprabasal cells that are not SCs. Analysis of published single-cell RNA sequencing data [27], indicated that putative YAP target genes are expressed by K15^+^ cells in the developing human cornea (Fig. S1B). Furthermore, the detection of nYAP in K15^+^ basal epidermal SCs suggests a broad role of YAP in epithelial SCs (Fig. 1A). To comprehensively study the role of YAP in SCs, we established primary human limbal epithelial culture [28]. At passage 1, the majority (~90%) of the cells expressed putative limbal SC/progenitor cell markers (P63, K15, IFITM3, CD63) [23] when grown on defined keratinocyte media without feeder cells (Fig. S1C). Next, limbal cells were grown on mitotically inactive (mitomycin treatment) NIH3T3-J2 fibroblasts, which are used to support LSC self-renewal. Of note, nYAP was inspected in proliferating stem/progenitor cells, often found at the peripheral zone of the colonies, while K15^-^ and P63^-^ differentiated cells frequently found at the colony center, express cytosolic YAP (cYAP) (Fig. 1B and Fig. S1D). A similar association between nYAP and stem/progenitors was confirmed in epidermal SC culture (Fig. 1B). Upon phosphorylation of amino residue S127, YAP becomes cytoplasmic, unable to carry out its putative nuclear co-transcriptional activities [29, 30]. Using a specific antibody raised against the phosphorylated form of YAP (pYAP), we confirmed that pYAP is preferentially found in the cytosol of differentiated cells (Fig. 1C, white arrows). This data suggests that nYAP localization and activity is linked with the undifferentiated cell state and that cYAP and pYAP are linked with cell differentiation in primary human limbal and foreskin cells. To further explore the link between YAP and differentiation, we explored YAP/pYAP expression before and after calcium-induced LSC differentiation [28]. Limbal epithelial cells were grown in a defined medium containing 150 μM (low) calcium to minimize differentiation (D0) or induced to differentiate in medium containing 1.2 mM (high) calcium for 7 days (D7). Immunofluorescent staining and quantitative real-time polymerase chain reaction (qPCR) confirmed successful cell differentiation hallmarked by the reduction in the levels of stem/progenitor cell markers (K15, P63) and increased expression of differentiation markers (K3 and K12) (Fig. 1D, F). Here too, nYAP was predominantly detected in undifferentiated K15^+^ cells, differentiation was correlated with a switch to cytosolic pYAP (Fig. 1D, E).

To investigate the influence of YAP on stemness, we performed knockdown experiments using endonuclease-digested silencing RNA (known as esiRNA) that are considered to have fewer off-targets and higher efficiency [31, 32]. As expected, the esiRNA had no effect on housekeeping genes (*HPRT1*, *ACTB*) (Fig. 1G) whereas YAP repression was efficient and resulted in the loss of stemness phenotype (Figs. 1H,I, S2A). To exclude the possibility of non-specific reagent activity, knockdown was performed with 2 conventional previously validated silencing RNAs against YAP (siYAP) [14] or with the pharmacological inhibitor of YAP, Verteporfin [33–35]. In agreement, both siRNA repression (Fig. S2B,C) and pharmacology inhibition (Fig. S2D) efficiently repressed YAP and stemness. Moreover, YAP knockdown by esiRNA or inhibition by Verteporfin (VP) drastically affected the colony formation efficacy of limbal epithelial cells (Fig. 1J–L), altogether, suggesting that YAP plays a role in preserving long-term LSC proliferation.

### YAP inhibition perturbs LSC function in vivo

**Fig. 2:**
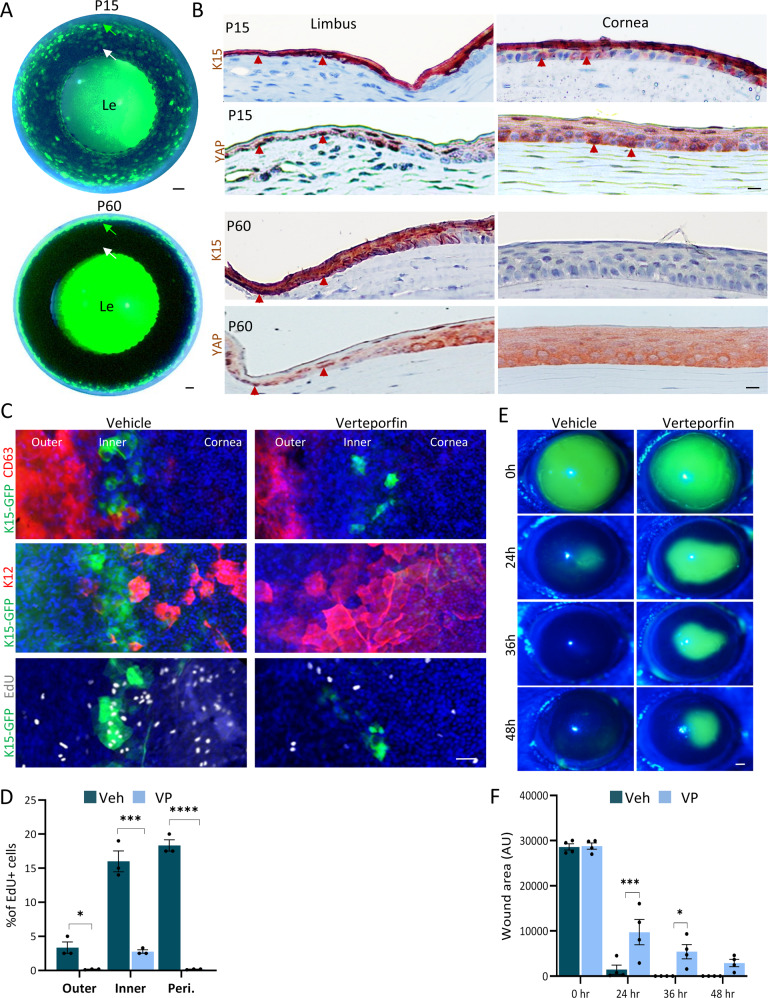
YAP inhibitor perturbs LSC function in vivo. **A** K15-GFP transgenic animals were sacrificed at P15 or P60 and the whole eye merged bright field and fluorescent images are shown. Green and white arrows indicate the limbus and cornea region, respectively. **B** Immunohistochemistry was performed on paraffin sections of the P15 or P60 wild-type mouse cornea using the indicated antibodies. Arrowheads indicate nuclear YAP or K15 expression in basal cells. **C**, **D** Daily sub-conjunctival injections (20 µl) of Verteporfin (20 µM) or vehicle (control) were performed for 4-days to adult K15-GFP transgenic mice. EdU was injected 6 h before eyes were enucleated and prepared for wholemount immunostaining. CD63 + outer quiescent LSCs, K15-GFP + inner active LSCs or K12 + corneal epithelial differentiated cells are shown, among them, EdU+ cells that are in mitosis and were quantified in (**D**) (*n* = 3 biological replicates). The basal layer was the focus point in the image acquisition. **E**,**F** The corneal epithelium of P60 mice was surgically removed using Algerbrush and animal were treated topically with Verteporfin (20 µM) or vehicle. At indicated time points, fluorescein dye staining was pictured (**E**) to follow epithelial healing and quantification is shown in (**F**) (*n* = 4 biological replicates). All data are represented as mean ± standard error of mean. Statistical significance was assessed by *t*-test (*, *p* < 0.05; ***, *p* < 0.001, ****, *p* < 0.0001). Immunohistochemistry and immunostaining data are representative from 3 to 5 biological replicates. Nuclei were detected by DAPI counterstaining. Scale bars are 500 µm (**A**) and the rest are 50 µm. Le Lens.

To validate the data and delineate YAP’s underlying role in LSC regulation in vivo, we characterized the expression pattern of YAP in murine cornea. Gene expression patterns and lineage tracing experiments implies that restriction of SCs to the limbus and renewal of the cornea by LSCs is established around post-natal day 15 (P15) and that the cornea fully matures by P60 [36–38]. Recent studies indicated that the limbus contains 2 clear LSC domains, the “outer” LSC zone which hosts LSC population that serve as a reservoir for wound healing whereas the “inner” LSCs actively renew the cornea [23, 39]. In line with previous reports [5, 23], transgenic K15-GFP serves as a useful indicator for active LSC and shows a punctuated pattern, whereas endogenous K15 protein labels the outer limbus. These apparent differences in the K15 transgene and endogenous protein could be due to differences in the location of the transgene in the genome or due to the lack of regulatory sites in the transgene. Nevertheless, these differences serve as a useful tool to identify LSC-like cells. Interestingly, in the immature P15 corneas, the LSC reporter transgene K15-GFP (Fig. 2A) and K15 protein (Fig. 2B) were detected in a punctuated pattern throughout both the limbal and central corneal epithelium, suggesting that at this stage undifferentiated cells are present in both limbus and cornea zones. By P60, however, the labeling pattern of K15-GFP and K15 (Fig. 2A,B) preferentially labeled the limbal epithelium and nYAP was predominantly expressed in the K15 positive regions (Fig. 2B). This expression pattern was corroborated by co-immunostaining with a different anti-YAP antibody (Abnova, H00010413-M01) indicating nYAP expression by adult murine K15 + basal limbal epithelial cells (Fig. S3A). To further explore the control of YAP in LSCs, we investigated the expression pattern of the Hippo pathway kinases, LATS1/2, which phosphorylate and inactivate YAP. Staining with antibodies that specifically recognize the active phosphorylated LATS1/2 (pLATS1/2) and pYAP, revealed that pLATS1/2 and pYAP are predominantly found in the corneal differentiation compartment at P60 (Fig. S3B,C). This observation suggests that in the cornea, LATS1/2 kinases become activated in the differentiated corneal cells and consequently phosphorylate YAP, thereby preventing its nuclear translocation.

To test YAP involvement with LSC function in vivo in the murine cornea, we performed sub-conjunctival injections (15 µl) of VP (20 µM) or vehicle (as control) once a day for 4 days. Analysis of cell and tissue morphology and apoptosis confirmed that the treatments with VP did not trigger cell loss or apoptosis (Fig. S4). As shown in Fig. 2C, the YAP inhibitor, VP, repressed markers of the outer (CD63) and inner (K15-GFP) LSCs and increased the differentiation marker, K12, in the limbus. Moreover, VP treatment decreased cell proliferation in both outer and inner limbus, as evidenced by lower incorporation of the nucleotide analogue 5-Ethynyl-2′-deoxyuridine (EdU) into the cells’ DNA (Fig. 2C,D) suggesting YAP plays a role in the control of proliferation of LSCs. To further explore the link between YAP and LSC functionality, we performed a large (2 mm) corneal epithelial debridement and followed epithelial healing with a fluorescein dye penetration test. As shown in Fig. 2E,F, VP significantly delayed wound healing, suggesting that YAP regulates LSC activity in vivo. Altogether, these data suggest that YAP regulates SC phenotype and cornea integrity in vivo.

### YAP inhibitor perturbs niche-mediated dedifferentiation of corneal committed cells

**Fig. 3:**
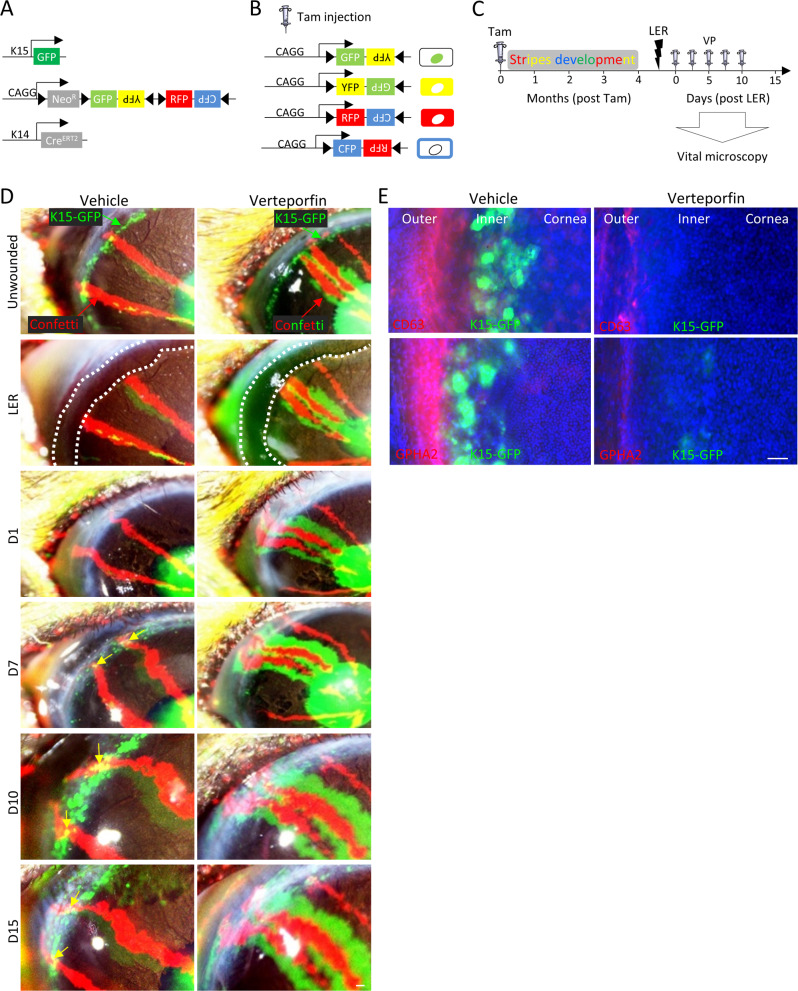
YAP inhibitor perturbs dedifferentiation. **A** Schematic illustration of triple transgenic animals (K15-GFP; Brainbow^2.1^; K14-Cre^ERT2^) used. **B** Examples of potential reorganization of the Brainbow cassette after tamoxifen induction. **C**–**E** As illustrated in (**C**), 2–3 month old triple transgenic animals were injected with tamoxifen (Tam) to induce the random and irreversible expression of Confetti reporters (**B**). **C**–**E** Four-months post Tam induction, fully developed Confetti + (RFP + ) limbal radial stripes were evident (see unwounded). Next, total limbal epithelial removal (LER, dotted white lines) performed by Algerbrush (see LER) and mice were sub-conjunctival injected with Verteporfin (VP, 20 µM) or control (Vehicle). For accurate clonal tracking over time, eyes with 2–3 RFP^+^ stripes were pictured in live animals (**D**). In vehicle treated mice, Confetti + (RFP^+^) corneal-committed cells repaired the denuded limbus by day 1 (D1) post LER and re-expressed K15-GFP by D10. In Verteporfin treated cornea, the recovery of K15-GFP was negligible. **E** On D15 post LER, eyes were enucleated and wholemount immunostaining for markers of the quiescent outer (GPHA2, CD63) LSCs or inner active (K15-GFP) LSCs are shown. Data represent 7 biological replicates. Nuclei were detected by DAPI counterstaining. Scale bars are 50 µm.

Catastrophic loss of the entire murine LSC population by surgical removal of the entire limbal epithelium is recovered owing to the high plasticity of corneal epithelial committed cells [5]. The latter cells repopulate the denuded limbus, and in case the niche is intact, they dedifferentiate into K15-GFP^+^ LSC-like cells and the corneal tissue integrity is maintained for many months. However, the involvement of YAP in this process is not known. To explore this hypothesis, the entire murine limbal epithelium (including marginal conjunctival and bordering peripheral corneal epithelium) was debrided, rendering the cornea deprived of LSCs. Total limbal epithelial removal (LER) was confirmed by histology (Fig. S5 and previous report [5]) and repopulation of the limbal epithelium by corneal committed cells that became nYAP-positive was already evident by day one post-injury (Fig. S5). The nYAP signal became even more pronounced and widespread by days 7–10, suggesting that YAP might be involved in the dedifferentiation process. To check this possibility and properly follow regeneration and the dedifferentiation processes in real-time, we used the triple transgenic K14-Cre^ERT2^; R26R-Brainbow^2.1^; K15-GFP mice [5]. In this multi-color “Confetti” lineage tracing system, upon transient exposure to tamoxifen (3–4 days injections) the K14-dependent Cre-recombinase induces the stochastic and irreversible labeling of K14 + limbal/corneal basal cells with one out of four fluorescent protein-coding genes (i.e., cytoplasmic red (RFP) or yellow (YFP), membrane cyan (CFP) or nuclear green (GFP)) [5, 20, 23] (Fig. 3A–C). Under homeostasis, the centripetal renewal of the corneal epithelium by Confetti+ LSCs can be visualized by intravital microscopy over time. Large Confetti+ limbal radial clones that emerged from the K15-GFP + inner limbus was evident 4 months post-induction (Fig. 3D, unwounded, yellow arrows mark double positive cells). As illustrated in Fig. 3C, LER was performed (dotted white lines), and tissue regeneration was traced following sub-conjunctival injection of Verteporfin or vehicle (as control). Injections were performed every other day until 10 days when recovery of K15-GFP signal peaked (see D10 in Fig. 3D), and at this point, treatment was ceased to avoid the potentially harmful effects of multiple eye injections. In all treatments, by day 1 (D1) post LER, stripes of corneal committed cells successfully repopulated the debrided limbal epithelium (Fig. 3D). Intriguingly, however, the K15-GFP recovery was markedly affected by Verteporfin treatment (Fig. 3D, see D7–15). Moreover, on D15 (5-days post last injection), the expression of LSC markers (K15-GFP, CD63 and GPHA2) was poorly revived in the limbus following Verteporfin treatment (Fig. 3D,E). This data suggests that YAP plays a vital role in the reprogramming of corneal-committed cells into LSC-like cells.

### Manipulation of stiffness impaired SC function and nYAP localization in vivo

**Fig. 4:**
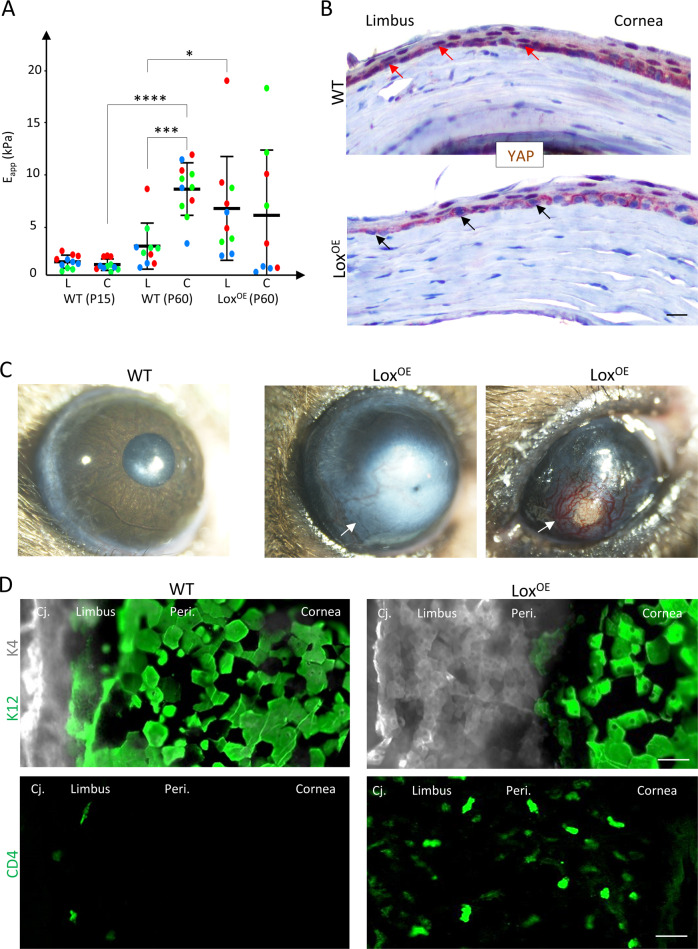
Manipulation of niche rigidity impairs SC phenotype and corneal integrity. **A** Atomic force microscopy measurement of the rigidity of the limbus (L) and the cornea (C) in wild type (WT) and Lox transgenic (Lox^OE^) mice at post-natal day 15 mice (P15) and P60 (the higher the Eapp the stiffer the tissue). Mean ± SD of the apparent Elastic modulus (in kPa). The plot shows the merge of three experiments performed on as many independent samples, using the same atomic force microscopy setup (deflection sensibility and cantilever spring constant). **B** Immunohistochemistry using anti-YAP antibody on paraffin sections of P60 cornea of the WT and Lox^OE^ mice. Red arrows indicate nYAP and black arrows indicate cYAP in the limbus region. **C** Bright field binocular images of the eyes of the WT and Lox^OE^ are shown. Lox^OE^ corneas often display peripheral and/or central opacification and neovascularization (white arrows). **D** Wholemount immunostaining for conjunctiva (K4), cornea (K12) and immune cell (CD4) markers of WT and Lox^OE^ cornea. Staining represents 3 biological replicates and statistical significance was assessed by *t*-test (*, *p* < 0.05; ***, *p* < 0.001, ****, *p* < 0.0001). Scale bars are 50 µm. Cj Conjunctiva, Peri Peripheral cornea.

Very little is known about the limbal niche formation and the involvement of biomechanical cues in this process. Previous measurements of the rigidity of the human cornea indicated that the human corneal center is much stiffer (20 kPa) than the limbus (8 kPa) [10]. We hypothesized that the differential stiffness develops at post-natal stage when the limbus becomes the SC niche. Yet, the rigidity of the murine limbus, whose anatomy slightly differs from that of the human limbus, has never been tested. To analyze the underlying rigidity that murine basal limbal/corneal layer cells may sense, the entire limbal/corneal epithelial layer was removed by ethylenediaminetetraacetic acid (EDTA) treatment, and the rigidity of the denuded surface was tested by atomic force microscopy (AFM). At P15, the stiffness of the murine cornea and limbus were comparable (~1 kPa) where SCs were uniformly scattered through the cornea (Fig. 4A). In the mature P60 murine, the limbus, although not as stiff as the human limbus, is a relatively rigid tissue (~2 kPa) while the corneal differentiation compartment is significantly stiffer (~7 kPa) (Fig. 4A). Together with the confinement of SCs to the limbus and the nYAP phenotype observed in that region (Fig. 2A,B), this data suggests that matrix stiffening might play an important role in LSC regulation.

To further explore whether matrix stiffening alone can induce differentiation, we investigated the corneas of adult transgenic animals that over-expressed the enzyme Lysyl oxidase (Lox) [40]. Lox catalyzes covalent crosslinking of collagen and elastin in the extracellular matrix (ECM), thereby increasing tissue stiffness [41–43]. AFM measurements revealed a ~2-fold increase in the stiffness of adult limbus of transgenic Lox (Lox^OE^) animals (Fig. 4A) that is coupled with abnormal detection of cYAP in the limbus, even at early disease stage, in zones that display normal by histology (Fig. 4B). Moreover, Lox^OE^ corneas often displayed typical hallmarks of a clinical entity known as “LSC deficiency” [44], including corneal opacification (Fig. 4B), abnormal presence of cells that express conjunctival cell markers in the cornea, and extensive infiltration of immune cells into the cornea (Fig. 4C). Taken together, this data implies LSCs can sense the stiffness of the niche matrix and biomechanical signal affects YAP localization and LSC function in vivo.

### Rigidity-induced adhesion and actomyosin contractility enhances cell differentiation through YAP inactivation

The association between the relatively low limbal rigidity, nYAP and stemness, suggests that corneal rigidity reduces nYAP to enhance differentiation. These relationships are inconsistent with many studies that linked nYAP with stiffer matrices and/or with activation of the RhoA pathway [14, 45, 46]. However, other studies have recently reported that lower rigidity or inhibition of the Rho-A pathway enhanced nYAP in mammary gland stromal fibroblasts [16], mouse incisor SCs [17], and muscle SCs [18], suggesting that cell context-specific factors modulate the response of YAP to substrate rigidity.

**Fig. 5:**
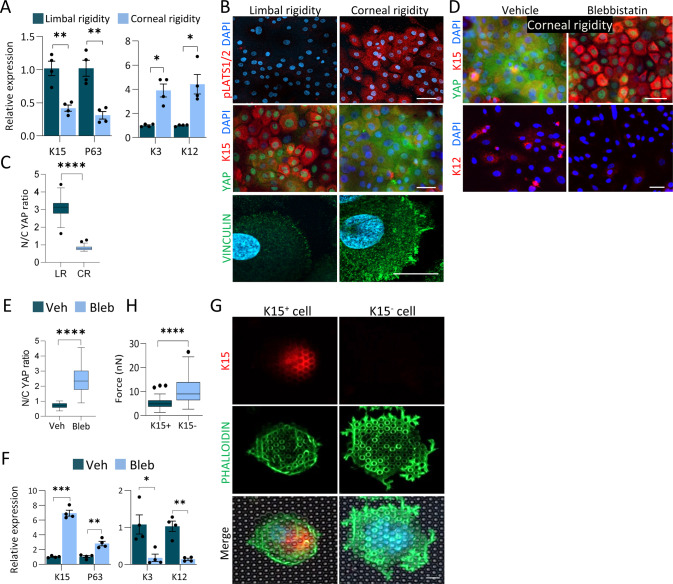
Corneal rigidity induces differentiation and inhibits nuclear localization of YAP. Primary human limbal epitheial cells were grown on a silicone substrate that mimics the rigidity of the human limbus (LR, 8 kPa) or cornea (CR, 20 kPa), coated with fibronectin and cultured for 4 days. Expression of stem/progenitor (K15, P63) or differentiation (K3, K12) genes was tested by quantitative real time PCR (qPCR) (**A**) or immunostaining with the indicated antibodies (**B**) and mean nuclear-to-cytoplasmic YAP intensity ratio was quantified (**C**). **D**–**F** Primary limbal epithelial cells were grown on silicone substrate that mimics corneal rigidity, were cultured for 4 days with Blebbistatin (Bleb) or Vehicle (Veh), the expression of the indicated markers was tested by immunostaining (**D**), mean nuclear-to-cytoplasmic YAP intensity ratio was quantified (**E**) and the expression of the indicated genes was tested by qPCR (**F**). **G** Primary human limbal epithelial cells were seeded on pillars that mimic limbal or corneal rigidity overnight. Immunostaining of K15 and F-actin (phalloidin) is shown, and measurements of forces generated by K15-positive and K15-negative cells (undifferentiated and differentiated; see Methods) are shown in (**G**,**H**), *n* = 44 and 52 cells respectively from three independent experiments. The mean nuclear-to-cytoplasmic YAP intensity ratio in (**C**, **E**) is shown by Tukey box-and-whisker. The force generated by LSCs is shown in (**G**) by Tukey box-and-whisker plot followed by Mann–Whitney test. The qPCR data were normalized to the housekeeping gene and is presented (mean ± standard error of mean, *n* = 4 biological replicates) as fold increase compared to control sample and statistical analysis was performed by *t*-test (*, *p* < 0.05; **, *p* < 0.01; ***, *p* < 0.001, ****, *p* < 0.0001). Immunostaining data are representative from 3 biological replicates. Nuclei were detected by DAPI counterstaining. Scale bars are 5 µm (**G**) and the rest are 50 µm.

As a control experiment, we first used two fibroblast cell lines, mouse embryonic fibroblasts (MEFs) and WI-38, which were expected to display “standard” YAP responses to rigidity. We grew both cell lines on substrates with stiffness ranging from 0.25 to 35 kPa and found a positive correlation between rigidity and nYAP (Fig. S6A,B). To further test the effect of substrate rigidity on YAP localization, primary human LSCs were cultivated for 4-days on substrates that recapitulate the rigidity of the human limbus (8 kPa, referred to hereafter as “limbal rigidity”) or cornea (20 kPa, “corneal rigidity”) [10]. In line with a previous report [13], growth on corneal rigidity-like gels decreased the expression of stem/progenitor cell markers (K15, P63) and increased differentiation markers (K3, K12) (Figs. 5A,B, S6C–E), as compared to control gels (limbal rigidity). In agreement with the in vivo data in mice (Fig. 2B, Fig. S3B,C), human LSCs grown on the corneal rigidity for 4-days displayed much higher levels of pLATS1/2 and cYAP (Fig. 5B,C). The stiffer substrate enhanced focal adhesion maturation as evidenced by Vinculin staining which is in line with previous observation in the cornea in wounding conditions [47] (Figs. 5B, S7A) and cell spreading (Fig. S7C), altogether suggesting that LSC differentiation is linked to activation of the mechanotransduction pathways. Next, we tested whether the rigidity-dependent YAP translocation and cell differentiation require actomyosin contractility. To this end, cells were seeded on matrices with corneal stiffness and actomyosin-mediated forces were inhibited by Blebbistatin (20 μM) for 4-days. Indeed, Blebbistatin attenuated the stiffness-induced transition to mainly cYAP (Fig. 5D,E), attenuated cell differentiation (Fig. 5D, F) and cell spreading (Fig. S7B). Collectively, these data suggest that stiffness-induced differentiation is controlled by LATS1/2 mediated cYAP localization through actomyosin contractility.

We next turned to characterize the contractile forces that the LSCs produce for mechanosensing. To that end, we used arrays of fibronectin-coated elastic polydimethylsiloxane (PDMS) pillars with limbal or corneal rigidity [48, 49]. After overnight incubation, the cell size was significantly smaller on the pillars with limbal rigidity and a clear inverse correlation between cell area and K15 expression was evident (Fig. S7C,D). Moreover, larger K15-negative differentiated cells typically displayed filamentous actin (F-actin) distributed as rings around individual pillars at the cell edge; in contrast, smaller K15 + cells typically showed much fewer F-actin rings, and rarely at the edge (Fig. 5G). F-actin around pillars is positively correlated with the level of the force on the pillars [50]; indeed, the cells that lacked K15 expression generated on average almost two fold higher forces on individual pillars compared to cells that had high K15 expression (Fig. 5H). This observation is in line with the formation of larger adhesions on the stiff matrix (Figs. 5B, S7A), which support the transmission of higher contractile forces. Altogether, these data suggest that matrices with limbal rigidity support the formation of small adhesions and weaker contractile forces that render low LATS1/2 activity and consequently favor stemness through maintaining nYAP.

### SMAD2/3 represses nYAP and mediates stiffness-induced differentiation

**Fig. 6:**
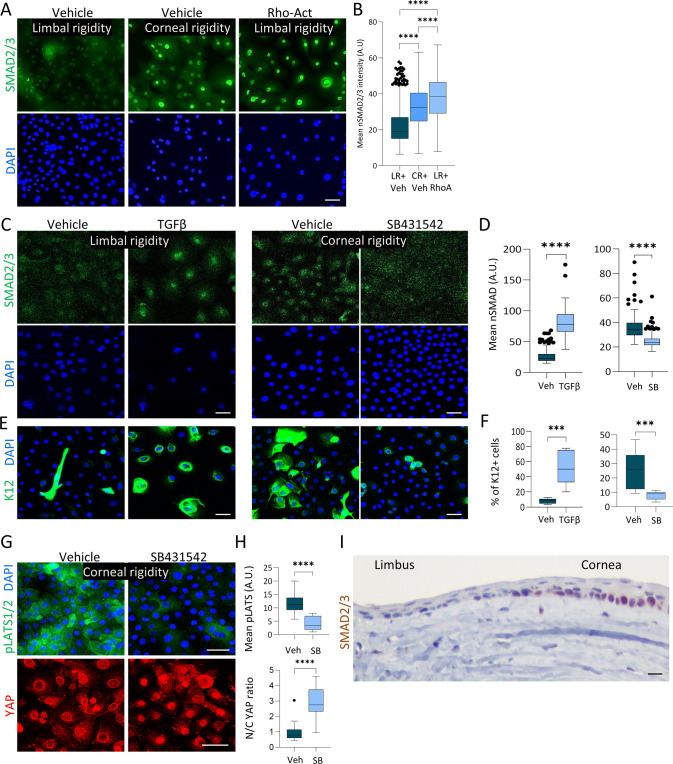
Stiffness-induced differentiation is mediated by SMAD2/3. **A**–**H** Primary human LSCs were grown on limbal or corneal rigidity and treated with Rho-Activator (Rho-Act) or Verteporfin (VP) for 4 days whereas treatment with TGFβ ligand or TGFβ pathway inhibitor (SB431542), or with control vehicle for 2 days. Cells were then fixed and immunostained with the indicated antibodies (**A**, **C**, **E**, **G**) and quantification of mean nuclear SMAD2/3 (nSMAD) (**D**), % of K12 + cells (**F**), or mean nuclear-to-cytoplasmic YAP intensity ratio and mean pLATS1/2 intensity is shown (**H**). **I** Immunohistochemistry of SMAD2/3 was performed on paraffin sections of the P60 mouse cornea. The regions of the limbus and corneal periphery are shown. Mean nuclear-to-cytoplasmic YAP intensity ratio, mean pLATS1/2 intensity, and mean nuclear SMAD2/3 intensity is shown by the Tukey box-and-whisker plot followed by Mann–Whitney test. % of K12 positive cells is shown by Tukey box-and-whisker plot followed by *t*-test with Welch’s correction (*, *p* < 0.05; **, *p* < 0.01; ***, *p* < 0.001, ****, *p* < 0.0001). Immunostaining, and immunohistochemistry, data are representative from 3 biological replicates. Nuclei were detected by DAPI counterstaining and scale bars are 50 µm.

As compared to a few other tissues, the human limbus is relatively stiff (8 kPa) whereas the cornea is stiffer (20 kPa). We, therefore, suspected that the very stiff corneal rigidity stimulates another signaling pathway that overrides the “standard” mechanotransduced nYAP location, thereby leading to cYAP localization. Recent studies have shown that SMAD2/3 inhibition attenuates epithelial cell differentiation and that there is synergy between myosin II and TGF-β in the regulation of SC differentiation [51, 52]. Typically, active phosphorylated SMAD2/3 proteins translocate to the nucleus, where they regulate transcription [53]. Indeed, nuclear SMAD2/3 were preferentially detected following calcium-induced differentiation of LSCs on plastic (Fig. S8) or following 4-days growth on corneal rigidity. Treatment with Rho Activator II (Rho-Act; 1 µg/ml) which is known to enhance the mechanotransduction pathway [54–56] was sufficient to induce nuclear SMAD2/3 in limbal rigidity (Fig. 6A,B).

Activation of SMAD2/3 with recombinant TGFβ on limbal rigidity sufficiently induced cell differentiation whereas inhibition of the TGFβ pathway by SB431542 on corneal rigidity reduced nuclear SMAD2/3 and inhibited differentiation (Fig. 6C–F). Importantly, the TGFβ pathway inhibitor reduced pLATS1/2 and enhanced nYAP (Fig. 6G,H), suggesting an inverse regulation between TGFβ/SMAD2/3 and YAP. Finally, immunohistochemistry demonstrated that in line with in vitro human cells, the nuclear SMAD2/3 signal is inconspicuous in the limbus, whereas it is prominent in the murine cornea in vivo (Fig. 6I). Altogether, these data suggest that the very stiff corneal rigidity induces SMAD2/3-mediated repression of nYAP to induce differentiation.

### Inhibition of mechanosensing and TGFβ pathway attenuates LSC differentiation on plastic

**Fig. 7:**
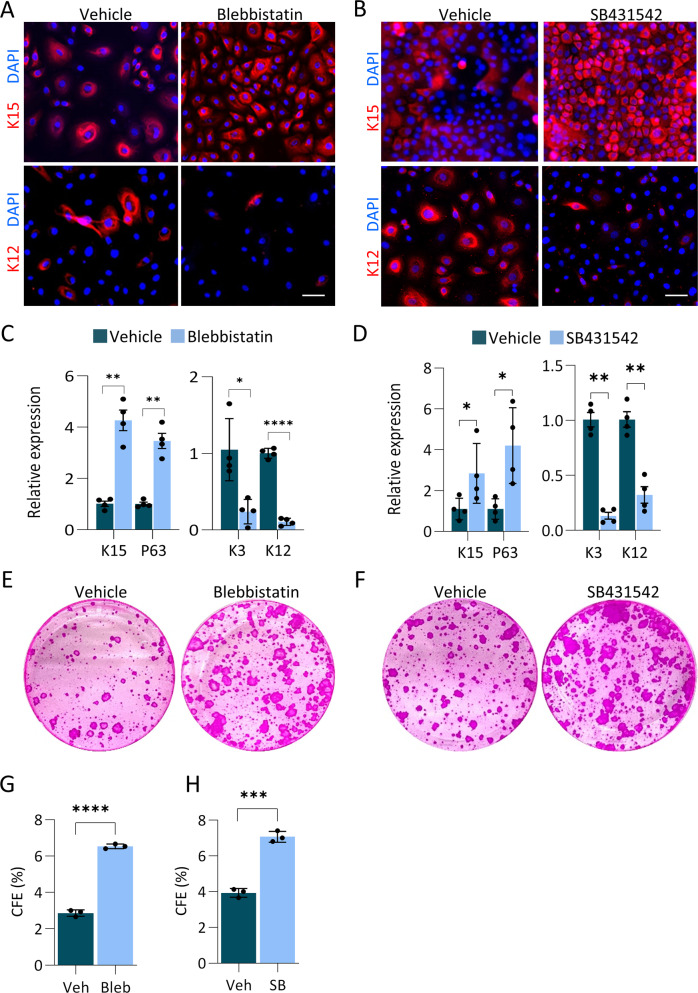
Inhibition of actomyosin contractility or TGFβ pathway attenuates LSC differentiation on plastic. **A**–**H** Primary human LSCs were grown on plastic and treated with Blebbistatin (Bleb) or TGFβ pathway inhibitor (SB431542) or vehicle (control) for 4 days and the expression of the indicated markers was tested by immunostaining (**A**,**B**), quantitative real time PCR analysis (**C**,**D**) or cells were subjected to clonogenicity assay and colonies were visualized by Rhodamine stain (**E**,**F**) and the number of the colony was quantified (**G**,**H**) (*n* = 3). Real-time data was normalized to the housekeeping gene and is presented (mean ± standard error of mean, *n* = 4 biological replicates) as fold increase compared to control sample and statistical analysis was performed by *t*-test (*, *p* < 0.05; **, *p* < 0.01; ***, *p* < 0.001, ****, *p* < 0.0001). Immunostaining, data are representative from 3 biological replicates. Nuclei were detected by DAPI counterstaining. Scale bars are 50 µm.

Finally, our results led us to consider the fact that plastic dishes that possess extremely high stiffness (Giga Pascals) are widely used to grow SCs for research and cell therapy. Therefore, we examined the impact of inhibition of mechanosensing by Blebbistatin or inhibition of the TGFβ pathway by SB431542 on plastic dishes. Primary LSCs were cultivated with each inhibitor for 4-days before harvesting and analysis. Treatment with either of the inhibitors resulted in enhanced expression of LSC markers, and reduced differentiation gene expression by immunostaining (Fig. 7A,B) and qPCR (Fig. 7C,D). Moreover, these inhibitors significantly augmented colony-forming capacity (Fig. 7E–H). Taken together, we conclude that SC culture on materials that extensively differ in the biomechanical properties of the native niche may affect SC self-renewal and long-term proliferative potential, and manipulating mechanosensing pathways may, at least partially, rescue this undesired effect.

## Discussion

SCs can sense the external environment and respond by making cell fate decisions to self-renew or differentiate. A relatively diminutive movement away from the niche to the differentiation compartment (e.g., of 20–30 microns) sufficiently induces the commitment to the differentiation of Lgr5-GFP + gut [57], K15-GFP + hair follicle and limbal SCs [5, 23, 58]. The importance of the niche in reprogramming is also striking. In response to SC depletion, epithelial cells that have already undergone commitment to differentiation, heal the affected niche, which stimulates their dedifferentiation into SC-like cells [59–61]. Mechanotransduction pathways and/or YAP may be involved in this unexpected cell plasticity [62]. To “read” and respond to the rigidity of the underlying matrix, SCs continuously probe the microenvironment and especially the underlying ECM by applying forces, engaging the Rho/actomyosin mechanotransduction pathways.

The influence of YAP on differentiation appears to be cell context dependent. YAP plays a positive role in the maintenance of adult SCs [46, 63–68] in some cell types, whereas YAP activity was linked with differentiated cells grown on stiff matrices [14, 45, 46]; in those cases, nYAP was correlated with adhesion growth [45] and large cell areas [14]. In line with previous reports [13, 24, 25], we observed that the higher rigidity of the cornea induces LSC differentiation. The present study, however, supports a role for YAP in the maintenance of an undifferentiated state. The inhibition of YAP in LSCs is induced by seeding LSCs on a substrate with corneal stiffness, or by TGFβ pathway induction (or by siRNAs or Verteporfin), all of which induced cell differentiation. The role of YAP in controlling stemness is supported by the observation that (i) nYAP was mainly inspected in LSCs under all conditions (murine, human in vivo, in vitro) and drastically decreases in differentiated cells. (ii) YAP inhibition by siRNAs or pharmacological inhibitor enhanced differentiation phenotype, (iii) attenuated LSC function in vivo (mouse) and in vitro (human) and (iv) impaired wound healing response in mice. Theoretically, some of the YAP-dependent roles described in the present study could be secondary to the decrease in cell density changes in vitro. However, a similar role for YAP was found in the in vivo experiments where cell densities are comparable. We, therefore, conclude that although cell density may influence YAP localization, nYAP is linked with stemness irrespective of cell density. The subtle difference in the levels of cYAP in differentiated human (lower or absent*)* and murine (detectable) cells, suggests that the degradation rates of cYAP may differ between species and/or conditions (in vivo vs in vitro). Interestingly, nYAP was not limited to LSCs but was also observed in some limbal and corneal superficial cells, suggesting that YAP may also play a role in terminal differentiation.

YAP’s importance in the eye and corneal development was evident as single-copy deletion of the *Yap1* gene in mice induced complex ocular abnormalities, including microphthalmia, thinner Descemet’s membrane, and corneal fibrosis, the latter phenotype being reminiscent of LSC deficiency [69]. The coincided stiffening and differentiation of the cornea compartment, imply that mechanosensing processes play a role in tissue development. To further dissect the role of YAP in corneal development, homeostasis and regeneration, future studies should conditionally ablate *Yap1* and YAP-related genes, in a tissue and stage-specific manner. According to the current dogma, YAP is typically bound to TAZ and both YAP/TAZ cannot bind to the DNA by themselves, they require additional factors to exert their function on gene expression. Particularly of interest, YAP has been shown to directly bind and regulate P63 expression in skin keratinocyte SCs [70] and adult lung basal SCs [71], and is implicated in regulating the transcription of SOX2 [72]. Since SOX2 and P63 interact and control LSC function [28], YAP may bind to P63/SOX2 to mediate proliferation.

A better understanding of YAP upstream regulators is needed to illuminate the complexity of mechanotransduction pathways and the differential responsiveness of different cell types to a range of substrate rigidities. Very stiff tissue rigidity, for example, the rigidity of the cornea, might stimulate non-conventional responsiveness to YAP localization via another signaling pathway that overrides the “standard” mechanotransduction signaling of nYAP location, thereby leading to cYAP localization. The present study suggests that corneal stiffness-induced differentiation involves the activation of SMAD2/3 and repression of YAP. The mechanism may involve the TGFβ ligand-mediated phosphorylation of SMAD2/3 that drives its nuclear localization and induces the transition to a differentiation regulatory network. TGFβ is known to bind a latency-associated peptide and the latent TGFβ binding-protein-1 [73] and αvβ6 integrin-dependent stiffness-induced contractility has been shown to liberate TGFβ in epithelial cells [74]. Interestingly, αvβ6 is also expressed by corneal epithelial cells [75] and they were also found to secrete latent-TGFβ-binding protein through extracellular vesicles [76].

An additional open question involves the regulation of niche rigidity. Do LSCs themselves regulate the stiffness of their underlying niche? Is there a key role for niche cells, vasculature, ECM protein composition in regulating the biophysical property of the niche? This control may involve the modulation of LOX family enzymes as well as the secretion of MMPs. While few recent studies focused on the biomechanics of epithelial SCs in vivo [9], the mechanobiology of stromal mesenchyme represents another frontier. The question of how do fibroblasts, immune, and other niche cells sense and respond to mechanical strain is open. Interestingly, changes in ECM stiffness can modulate the morphology, cytoskeletal organization, and subcellular pattern of force generation in corneal stromal cells treated with TGF-β1 [77].

The upstream regulation of YAP may be complex and involve the integration of diverse signals. The activation of LATS1/2 by stiff matrix coincided with YAP phosphorylation and cytoplasmic localization. However, LSCs that were co-cultured with NIH-3T3-J2 feeder cells on a plastic dish (Giga Pascals) expressed high levels of nYAP in the colony periphery (Figs. 1B, S1D), suggesting that feeder cells provide an essential signal that overrides mechanobiological control of YAP localization and stemness. Indeed, the induction of differentiation by calcium (on a plastic dish without feeder cells) was more forceful than differentiation induced by stiffer gel (compare Fig. 1F with Fig. 5A).

In conclusion, this study suggests that biomechanical signals provide a critical cell fate determination signal to LSCs under homeostasis and regeneration. It will be of interest to investigate the relevance of these findings to corneal pathologies, such as LSC deficiency or keratoconus, that may involve changes in ECM rigidities [13, 78]. While this study provides a potential explanation for the positive effect of ROCK and TGFβ inhibitors on the long-term expansion of epithelial cells [52], further study of LSC mechanotransduction pathways is needed to improve LSC expansion, allowing their optimal application in regenerative medicine.

### Reporting summary

Further information on research design is available in the Nature Portfolio Reporting Summary linked to this article.

## Supplementary information

SUPPLEMENTAL MATERIAL

Reporting summary

## Data Availability

All data needed to evaluate the conclusions in this study are presented in this paper or the supplementary information. The materials described in this study are either commercially available or available upon reasonable request from the corresponding authors.
