# Supplementary material for: The biophysical property of the limbal niche maintains stemness through YAP
Source: Cell Death Differ. 2023 Apr 24;30(6):1601–14. doi: 10.1038/s41418-023-01156-7 (PMC10244376; doi:10.1038/s41418-023-01156-7)
Supplement: Supplementary file 1 — SUPPLEMENTAL MATERIAL [file 41418_2023_1156_MOESM1_ESM.docx]

**­­­­­­­­­­­­­­­­­ Supplementary information**

**The biophysical property of the limbal niche maintains stemness through YAP**

Swarnabh Bhattacharya^1,2,3#^, Abhishek Mukherjee^1^, Sabrina Pisano^4^, Shalini Dimri^1^, Eman Knaane^1^, Anna Altshuler^1^, Waseem Nasser^1^, Sunanda Dey^1^, Lidan Shi^1^, Ido Mizrahi^1^, Noam Blum^1^, Ophir Jokel^1^, Aya Amitai-Lange^1^, Anna Kaganovsky^1^, Michael Mimouni^5^, Sergiu Socea^5^, Mohamad Midlij^6^, Beatrice Tiosano^6^, Peleg Hasson^1^, Chloe Feral^4^, Haguy Wolfenson^1*#^, Ruby Shalom-Feuerstein^1*#^

*Equal contribution and ^#^correspondence

(SB swarnabh_bhattacharya@dfci.harvard.edu, HW [haguyw@technion.ac.il](mailto:haguyw@technion.ac.il), RSF [shalomfe@technion.ac.il](mailto:shalomfe@technion.ac.il))

^1^Department of Genetics & Developmental Biology, The Rappaport Faculty of Medicine & Research Institute, Technion Integrated Cancer Center, Technion - Israel Institute of Technology, 31096 Haifa, Israel; ^2^Department of Medical Oncology and Center for Functional Cancer Epigenetics, Dana–Farber Cancer Institute, Boston, MA 02215, USA. ^3^Departments of Medicine, Brigham & Women’s Hospital and Harvard Medical School, Boston, MA 02115, USA: ^4^Université Côte d’Azur, INSERM, CNRS, IRCAN, 06107 Nice, France; ^5^Department of Ophthalmology, Rambam Health Care Campus, 31096 Haifa, Israel. ^6^Department of Ophthalmology, Hilel Yafe Medical Center, Hadera, Israel.

**Methods**

**Figure S1. Characterization of human limbal epithelial cells *in vivo* and *in vitro***

**Figure S2.** **Knockdown of YAP by silencing RNAs or verteporfin induces LSC differentiation.**

**Figure S3.** **Analysis of YAP and Hippo pathway kinases.**

**Figure S4.** **Verteporfin did not elicit cell toxicity.**

**Figure S5. Analysis of YAP expression following limbal epithelial depletion.**

**Figure S6. Corneal rigidity induces differentiation of human LSC.**

**Figure S7. Analysis of cell area and adhesions on gels and pillars.**

**Figure S8.** **Nuclear SMAD2/3 is associated with differentiation.**

**Supplementary Table S1. Sequences of qRT-PCR primers.**

**Supplementary Table S2. Source of antibodies used.**

**Methods**

***Animal handling***

Animal care and use conformed to the ARVO Statement for the Use of Animals in Ophthalmic and Vision Research and was approved by the local ethical committee (IL0980713). The mouse strains were kept at C57/BL6 background. K15-GFP (#005244), R26R-Confetti (#013731), K14-Cre^ERT^ (#005107) were from JAX (Bar Harbor, ME). Lox^OE^ was previously described (1). Cre recombinase activity was induced by injecting intraperitoneally (200 μl), 4 mg/day of Tamoxifen (T5648, Sigma, St. Louis, MO) dissolved in corn oil for 3-4 consecutive days, as previously reported (2). For wounding, mice were anesthetized (2% Isoflurane) and injected intramuscularly with analgesic Buprenorphine (0.03 mg/ml, 50 µl). Limbal epithelial removal and central corneal wounding (2 mm diameter) was performed using an ophthalmic rotating burr (Alger brush) under fluorescent binocular. The wounded corneas were stained (1% fluorescein) and the wounded area was imaged and quantified (NIS-Elements analysis D and ImageJ software). For sub-conjunctival injection, the bulbar conjunctiva was pulled using forceps and 15 μl of Verteporfin (Sigma, SML0534) was injected under the binocular using a 30-gauge needle connected to 1 mL syringe. Slow injection into the space between the conjunctiva and the sclera was performed to create a ballooning effect in the peri-limbal conjunctival zone. The injections were made on alternate days for the nasal and temporal sides. Sample sizes were determined according to the standard protocols in the field. No criteria were set for excluding mice and no blinding to group allocation was performed.

***Cell culture, differentiation, transfection, and Real-time PCR***

Human limbal rings from cadaveric corneas were obtained under the approval of the local ethical committee and informed consent was obtained from all subjects (0136-19-HYMC). Tissue were stored for usually for few days in corneal storage medium (Corneal Chamber, CTC 001-01, ALCHIMIA). The epithelium was separated from the underlying stroma following incubation with dispase II (GIBCO, Life Technologies, USA). Cells were cultured at 37C, 5% CO2, and 20% O2. For clonogenicity assay cells were grown in co-culture with mitomycinized growth-arrested J2-NIH 3T3 cells in Green medium (60% DMEM (GIBCO), 30% DMEM-F12 (GIBCO), 10% FCII serum (Hyclone), 1mM L-Glutamine (Biological Industries), 1mM Sodium Pyruvate (Biological Industries), 0.2mM Adenine (Sigma), 5mg/ml Insulin (Sigma), 0.5mg/ml Hydrocortisone (Sigma), 10mM Choleratoxin (Sigma), 10ng/ml EGF (Peprotec)) and split in 80% confluence. For efficient transfection and controlled calcium-induced differentiation, cells were switched to a defined medium with supplements (SCMK001, Millipore, United States) containing 1% penicillin/streptomycin and low calcium (150µM). Cells were seeded and grown to 80-100% confluency for differentiation and then switched to high (1.2mM) calcium for up to 1 week. Cells were grown on defined media and collected at indicated time points after treatment with vehicle or indicated factors Blebbistatin (para-nitroblebbistatin, Optopharma Ltd), or Verteporfin (Sigma, SML0534) and subjected to the Clonogenicity test. Colonies were counted by the automated measurement setting and the number of colonies was provided by the Nis-element D software. In all the experiments hLSC between passages 1-2 were used. Cells were assessed regularly for mycoplasma contamination using a MycoAlert PLUS Mycoplasma Detection Kit (Lonza, Basel, Switzerland).

MEFs and WI-38 cells were serum-starved for 15 hours and were kept in suspension in a serum-free medium for 30 minutes before seeding on fibronectin-coated PDMS gels to synchronize YAP localization to the cytoplasm. Cells were cultured in growth medium for 12 hours, fixed with 4% PFA, and then underwent immunostaining. Cells were imaged by ImageXpress® Micro Confocal (IXMC) microscope (Molecular Devices) with a 20X objective. Images were analyzed by the Translocation-Enhanced application module in MetaXpress® Software. In this module, an “inner region” shrunk in 0.5 µm from the detected nucleus was set to indicate the nuclear area, and an “outer region” expended out 0.5 µm from the detected nucleus was set to indicate the cytoplasmic area with a width of 2 µm. The Pearson’s correlation coefficient of the pixel intensity of YAP stain and nucleus stain in the two regions was calculated by the algorithm in the software. Correlation value of 1.0 indicates that the two stains overlap perfectly; –1.0 indicates complete lack of overlap between the two stains; and 0 indicates that the stains are independent. Cells with a correlation coefficient above 0.65 were considered YAP localized inside the nucleus. Nuclear-to-cytoplasmic (N/C) YAP average intensity was the average YAP intensity in inner regions divided by the average YAP intensity in outer regions.

For TUNEL, ApopTag® Red In Situ Apoptosis Detection Kit (S7165, Sigma) was used and staining were done according to the manufacturer’s instruction. For transfections, cells were seeded on plastic dishes and the next day transfected (Lipofectamine RNAimax, ThermoFischer) with 50nM esiRNA against EGFP (EHUEGFP, Sigma) or esiRNA against YAP (EHU113021, Sigma) or with following siRNA against YAP1 GACAUCUUCUGGUCAGAGA dTdT, YAP2 CUGGUCAGAGAUACUUCUU dTdT or ctl siRNA (Sigma, SIC001). Cells were collected 48-72 hrs after transfection. Real-time PCR analysis cells were washed with cold PBS and RNA was isolated using TRI-Reagent (Sigma) according to the manufacturers’ instructions. cDNA was prepared by reverse transcription polymerase chain reaction (RT-PCR) using the Qscript cDNA synthesis kit (Quantabio) according to the manufacturer’s instructions. Quantitative real-time polymerase chain reaction (qPCR) was performed with FastSYBR green master mix (Thermo). Samples were 40 cycled using StepOnePlus (Applied Biosystems) qPCR system. Relative gene expression was normalized to GAPDH and calculated according to the DDCT method for qPCR.

***Immunostaining***

For in-vitro staining, cultured epithelial cells or mouse fibroblasts (3,4) were grown on glass coverslips or silicone substrates and fixed in 4% paraformaldehyde (PFA) (Sigma) for 15 minutes and then permeabilized with 0.1% Triton X-100 (BioLab) in PBS for 10 minutes. Blocking was done with bovine serum albumin (Biological Industries), 3% donkey serum (Jackson) and 0.1% Triton X-100 for at least 30min. Following these treatments, cells were incubated for overnight with primary antibody at 4 degrees and further incubated with secondary antibodies (1:500) for 1 hour followed by 4′,6-diamidino-2-phenylindole (DAPI) or phalloidin 488 (1:400, Thermo Fisher, A12379) staining, and mounting (Epredia™ 9990402, Fisher Scientific).

For wholemount staining, the cornea was isolated and fixed (2% formaldehyde) for 2 hours, room temperature followed by permeabilized (0.5% Triton, 5 hours). Blocking was done for 1 hour (0.1% TritonX-100, 2% Normal donkey serum, 2.5% BSA) and then incubated with primary antibody overnight, 4°C on a shaker and further incubated with secondary antibodies (1:500, 1 hour), followed by DAPI, tissue flattening under a dissecting binocular and mounting (Thermo Scientific). For EdU staining, a Single intraperitoneal injection of 200 μl (7.5 mg/ml) EdU (Invitrogen) was performed and 6 hours later, tissues were processed. Isolated corneas were fixed (2% PFA, 1 hour) and stained (Click-iT, Invitrogen) according to the manufacturer’s instructions followed by wholemount staining protocol (described above) for other markers staining.

Paraffin sections (5µm) of mouse cornea and human corneas were used and processed for immunohistochemistry. Paraffin sections were deparaffinized by heating for 1 hour at 60°C, then rehydrated twice in Xylene for 5 minutes, followed by two incubations in 100% Ethanol for 5 minutes. Suppression of endogenous peroxidase activity was achieved by incubation in Methanol with 1% H_2_O_2_, incubation in 70% Ethanol for 2 minutes, followed by a rinse in Distilled water. Antigen retrieval was performed with an unmasking solution (Vector Laboratories, H3300), followed by blocking (10% goat serum), and incubation with a primary antibody (overnight, 4C), secondary antibodies (Universal Immuno peroxidase Polymer anti-rabbit/mouse, a ready-made solution, 1 hour) followed by substrate addition (AEC solution), Hematoxylin staining and mounting (Thermo Scientific).

Images were taken by Nikon Eclipse NI-E upright microscope and Zeiss LSM880 confocal microscope. For quantification, three to three to five different fields were imaged from different experiments and the indicated mean fluorescence intensity was calculated by ImageJ software. All the images were acquired at the same exposure time, magnification and fluorescence intensity for the control and experimental conditions.

***Tissue stiffness measurement by Atomic Force Microscopy (AFM)***

Eyes were collected from 2 and 6 weeks old mice (C57BL/6J or Lox^OE^) following CO_2_ sacrifice. Corneas with surrounding limbus were isolated as previously described (5), then treated for 30min in 2.5mM EDTA in PBS at 37C to allow epithelium removal. Next, samples were prepared by dissecting radially the tissue in two halves, each of them containing both the limbus and the cornea. The two halves were placed on SuperFrost Plus adhesion slides (Thermo Scientific) side by side and at 180 degrees to each other to have for both the areas of interest, the limbus and the cornea, free access to the sample for the AFM probe. The samples were allowed to adhere to the charged glass for 20’, then the fragment of glass containing the mounted tissues was cut out from the glass slide and glued on the bottom of a 50 mm dish (Willco Glass Bottom Dish). Before measurements the specimen was first rinsed and after covered with 4 ml of PBS 1x. After determining both the deflection sensitivity of the AFM system in PBS 1x using a clean glass slide fragment glued on Willco Glass Bottom Dish and the spring constant of the AFM cantilever by means of the thermal tune method, the sample was mounted on the AFM system and after thermal stabilization, for each limbus and the corresponding cornea (n=3), a minimum of 3 different areas were analyzed using the “Point and Shoot” method, collecting on average 100 force-distance curves at just as many discrete points spaced by at least 20 μm. Force-distance curves were collected on samples using a velocity of 2 μm/s, in relative trigger mode and by setting the trigger threshold to 1 nN. The apparent Young's modulus was calculated using the NanoScope Analysis 1.80 software (Bruker Nano Surfaces, Santa Barbara, CA, USA) applying to the force curves, after the baseline correction, the Hertz spherical indentation model using a Poisson’s ratio of 0.5. All the force-distant curves having a not clear base line, a maximum above or below 1nN or a change of slope in the region of the fitting (minimum and maximum force fit boundary 0% and 25%, respectively) were rejected and not considered for the analysis. Only the apparent Young’s modulus values corresponding to a fit with R^2^> 0.85 were considered for the analysis. The tissue mechanical proprieties were obtained by using a Bioscope Catalyst (Bruker Nano Surfaces, Santa Barbara, CA, USA), coupled with an inverted optical microscope (Leica DMI6000B, Leica Microsystems Ltd., UK). The force-distance curves needed to calculate the apparent Young’s modulus were collected using a Borosilicate Glass spherical tip (5 μm of diameter) mounted on a cantilever with a nominal spring constant of 0.06 N/m (Novascan Technologies, Ames, IA USA).

***Silicone substrates preparation***

To obtain substrates of different stiffness, the silicone elastomer CY52-276 (Dowsil) was used, with various ratios of the silicone base and crosslinking agent (components A/B): 1:1.8 for 8 kilo Pascal (kPa), and 1:2.5 for 20 kPa (6). The choice of this elastomer was due to its reported Young’s moduli that are within the range of the majority of mammalian tissues, in the lower kPa scale (7–9), as well as due to the uniform fibronectin adsorption between elastomers of different rigidities (10). The dimeric fibronectin adsorbed to the surface generates a thin coat thereby acting as a chemical ligand while the cells sense the stiffness of the underlying elastomer. After thorough mixing of both components, air bubbles were eliminated by application of vacuum for 30 min. Gels were spread on glass-bottom dishes followed by incubating at 70 °C for 2 h. Then the gels were sterilized by 30 min immersion in 70% ethanol. The substrate surfaces were coated with 10 μg/ml fibronectin for at least 1 h before seeding cells.

***Pillar arrays fabrication***

Pillar fabrication was done by pouring PDMS (Sylgard 184, Dow; mixing ratio – 10:1) into silicon molds (fabricated as previously described (11) with holes at fixed depths and distances. The molds were then placed, face down, onto glass-bottom 35 mm dishes (#0 coverslip, Cellvis) which were incubated at 65°C for 12h to cure the PDMS. The molds were next peeled off while immersed in ethanol to prevent pillar collapse. The ethanol was then replaced by serial dilutions with PBS, and human plasma full-length fibronectin (Merck) was added to the dish at a final concentration of 10 µg/µl for a 1h incubation at 37°C. Next, residual fibronectin was washed away by replacing the buffer to defined media with 20 mM HEPES.

All pillars had a diameter of 2 µm, and heights of 5.3 or 13.2 µm. We used 2 μm diameter pillars as these can be used to measure the long-term time-dependent forces that are generated after initial formation and reinforcement of the adhesions (12). The center-to-center spacing between pillars was 4 μm. Pillar bending stiffness, k, was calculated by Euler–Bernoulli beam theory:

$k= \frac{3}{64}\pi E\frac{D^{4}}{L^{3}}$

where D and L are the diameter and length of the pillar, respectively, and E is the Young’s modulus of the material (=2 MPa for the PDMS used here).

***Pillar displacement measurements***

For measuring forces by K15-positive and K15-negative cells, the cells were grown in low and high-calcium media, respectively, for 4 days. On the day of the experiment, the cells were trypsinized, centrifuged with growth medium, and then resuspended and pre-incubated in defined media with HEPES at 37°C for 30 min before plating them on the 5.3 µm fibronectin-coated pillars.

Time-lapse imaging of cells spreading on the pillars was performed using an inverted microscope (Leica DMIRE2) at 37°C using a 63x 1.4 NA oil immersion objective. Brightfield images were recorded every 10 seconds with a Retiga EXi Fast 1394 CCD camera (QImaging). The microscope and camera were controlled by Micromanager software (13). In all cases, we imaged cells that were not in contact with neighboring cells when plated on the substrates. For each cell, a movie of 1-3 hours was recorded. To minimize photodamage to the cells, a 600 nm longpass filter was inserted into the illumination path.

Tracking pillar movements over time was performed with ImageJ (National Institutes of Health) using the Nanotracking plugin, as described previously (11). In short, the cross-correlation between the pillar image in every frame of the movie and an image of the same pillar from the first frame of the movie was calculated, and the relative x- and y-position of the pillar in every frame of the movie was obtained. To consider only movements of pillar from their zero-position, we only analyzed pillars that at the start of the movie were not in contact with the cell and that during the movie the cell edge reached to them. Drift correction was performed using data from pillars far from any cell in each movie. For each pillar, the displacement curve was generated by Matlab (MathWorks).

***Statistical analysis***

Prism 9 (Graph Pad) was used for data plotting and statistical analysis. All experiments were performed independently at least three times. All quantifications represent the mean ± standard error of the mean (SEM) or as indicated in the legends. Images are representative of experiments that have been repeated independently at least three times. Data were tested for normality where applicable by the Shapiro-Wilk method. Group comparison was performed using a two-tailed unpaired Student’s *t* test or Mann-Whitney test as indicated in the legends. Multiple groups comparison was performed by ANOVA followed by Tukey’s test. All measurements were taken from distinct samples, and no data were excluded. Sample sizes were determined according to the standard protocols in the field. No randomization methods were used. Differences were considered to be statistically significant from a *p*‐value below 0.05.

**Supplementary Figures**

**
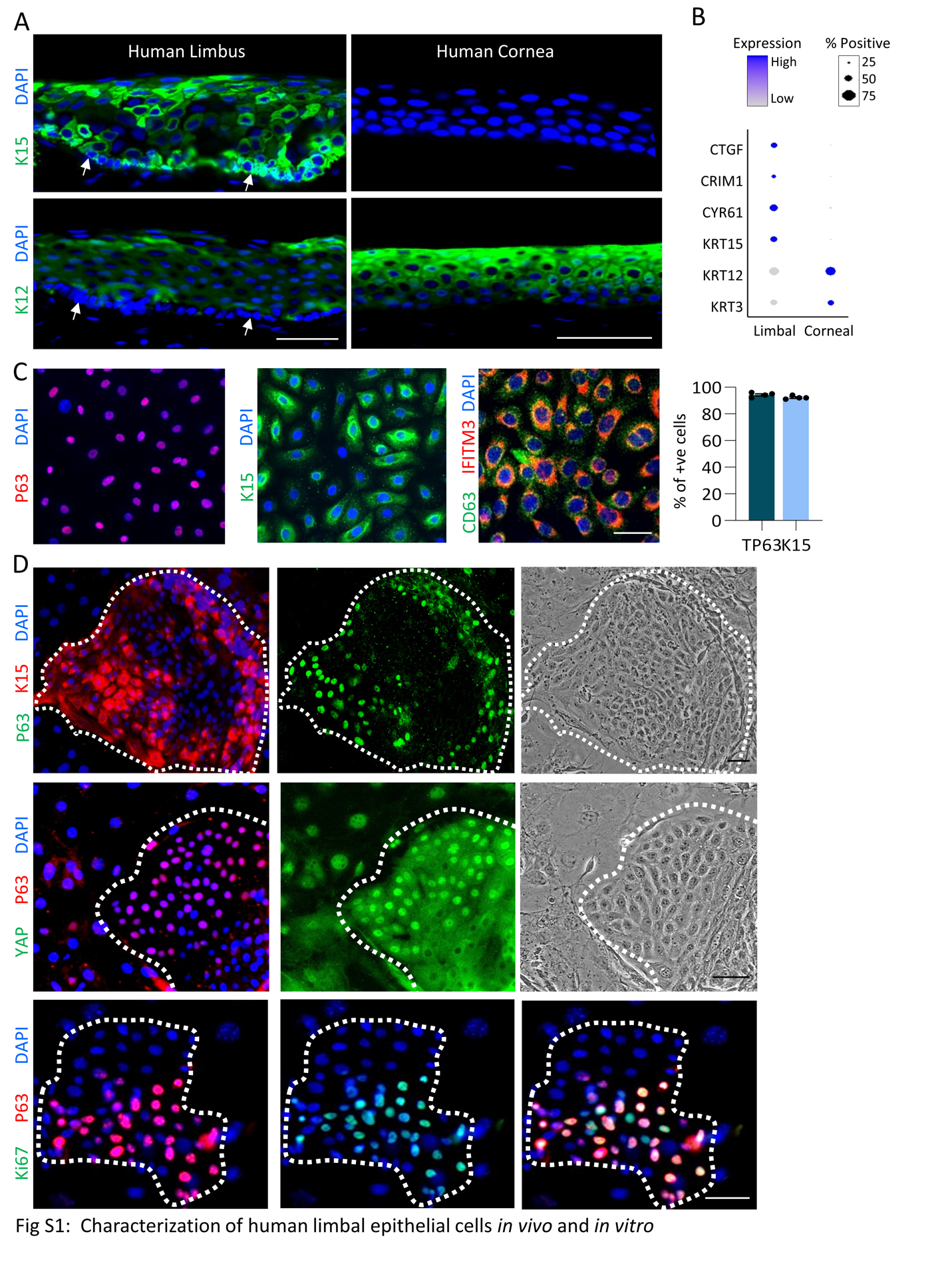
Figure S1. Characterization of human limbal epithelial cells *in vivo* and *in vitro.*** (A) Immunofluorescent staining of K15 and K12 in human limbal and corneal sections. White arrows indicate K15^+^ and K12^-^ basal limbal epithelial cells. (B) Analysis of single cell RNA sequencing of the developing human cornea at post conception week 17-18. Dot-plot map demonstrates that K15^+^/K3^-^/K12^-^ LSC-like cluster expresses putative YAP target genes (CTGF, CRIM1, CYR61) whereas the cluster of K15^-^/K3^+^/K12^+^ corneal differentiated cells does not. (C) Primary human limbal epithelial cells were grown on a plastic dish in low calcium media without feeder cells for 1 day and the expression of the indicated stem/progenitor markers was tested by immunostaining and quantified (right panel in C). (D) Primary human limbal epithelial cells were co-cultured with NIH-3T3-J2 feeder cells, grown on a plastic plate for 4 days and the expression of the indicated markers was examined by immunostaining. Immunostaining, data are representative from 3-4 biological replicates. Nuclei were detected by DAPI counterstaining. Scale bars are 100µm (A) and the rest are 50µm.


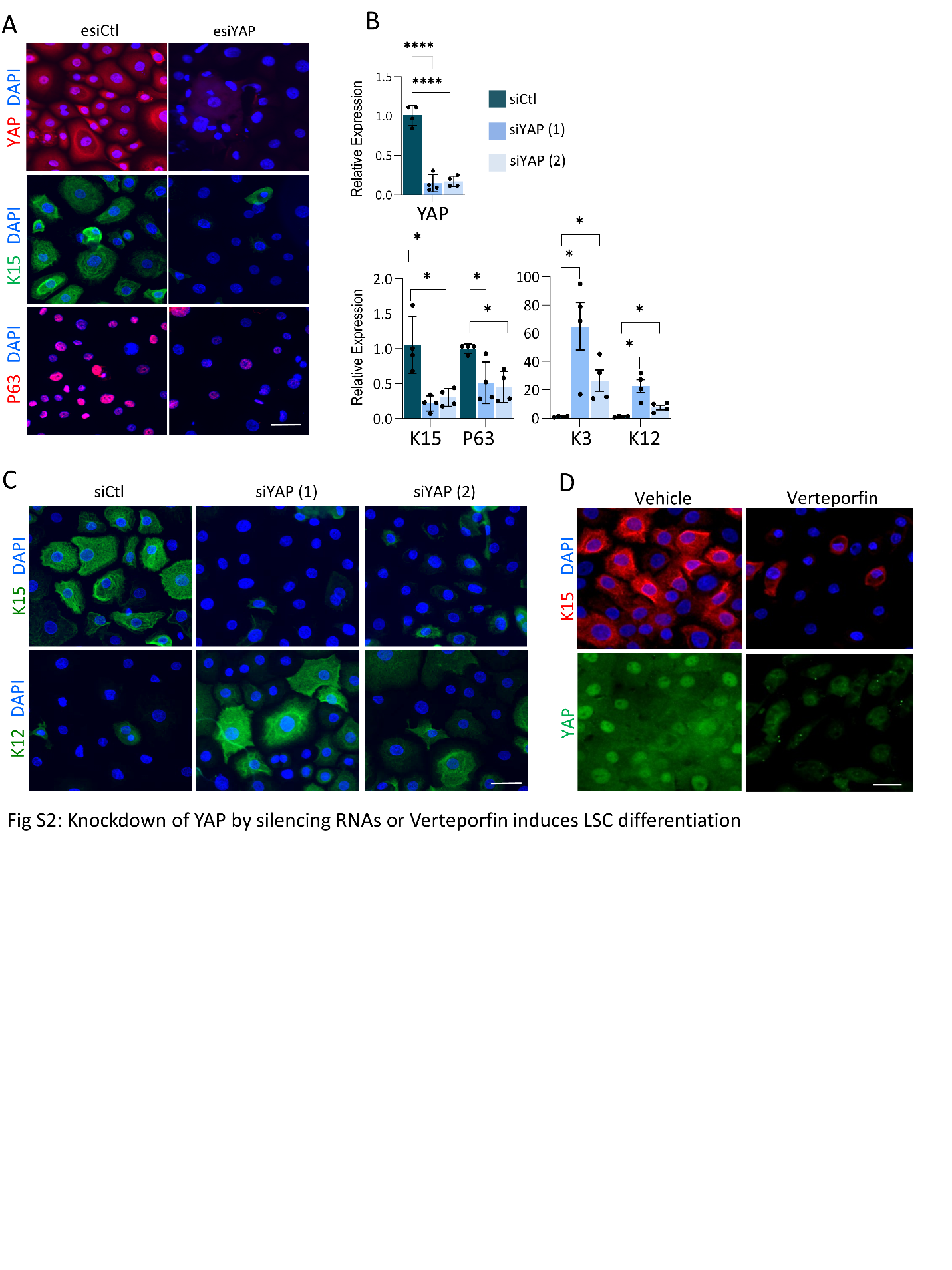
**Figure S2.** **Knockdown of YAP by silencing RNAs or verteporfin induces LSC differentiation.** (A-C) Primary human LSCs were grown on plastic dishes and transfected with esiRNA or two distinct silencing RNA sequences against YAP (siYAP) or with control sequence (siCtl). (A-C) The expression of the indicated genes was examined by real-time PCR analysis (B), or by immunofluorescent staining (A, C) 48-72 hours post-transfection. (D) Primary human LSCs were grown for 4-5 days on a silicone substrate that mimics the rigidity of the limbus coated with fibronectin in the presence of Verteporfin of Vehicle (control) and stained for K15 or YAP. Real-time PCR data were normalized to the housekeeping gene and is presented (mean ± standard error of mean, n=4 biological replicates) as fold increase compared to the control sample and statistical analysis was performed by t-test (*, p < .05; ****, p < .0001). Nuclei were detected by DAPI counterstaining. Scale bars are 50µm.


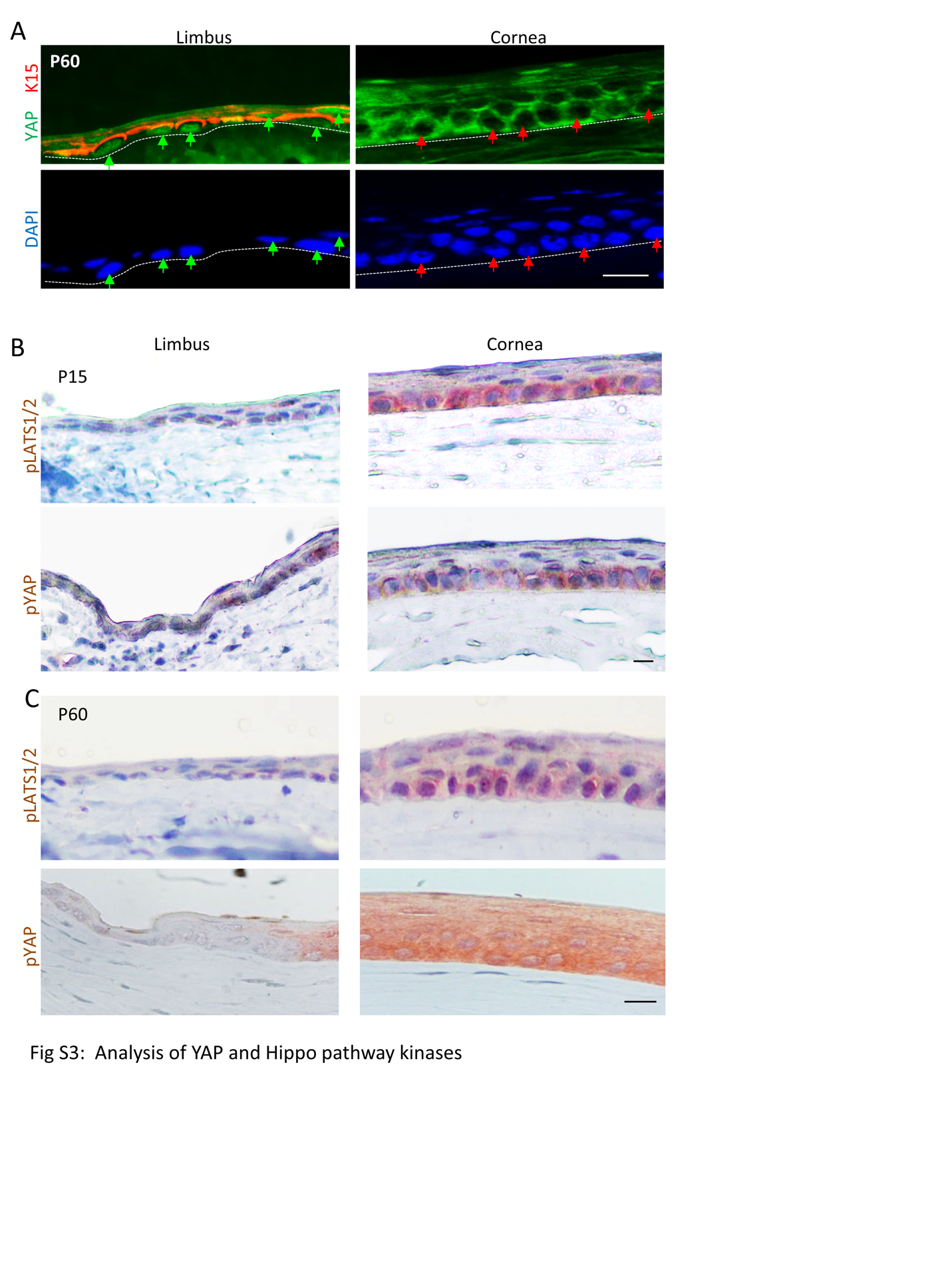
**Figure S3.** **Analysis of YAP and Hippo pathway kinases.** (A) Co-immunofluorescent staining of YAP and K15 in tissue sections of P60 murine cornea. Green arrows indicate nYAP in the limbus and red arrows indicate cYAP in the cornea compartment. (B-C) Immunohistochemistry of pYAP and pLATS1/2 was performed in the tissue sections of P15 (B) and P60 (C) of wild type murine cornea. Immunostaining, and immunohistochemistry data are representative from 3 biological replicates. Scale bars are 50µm.


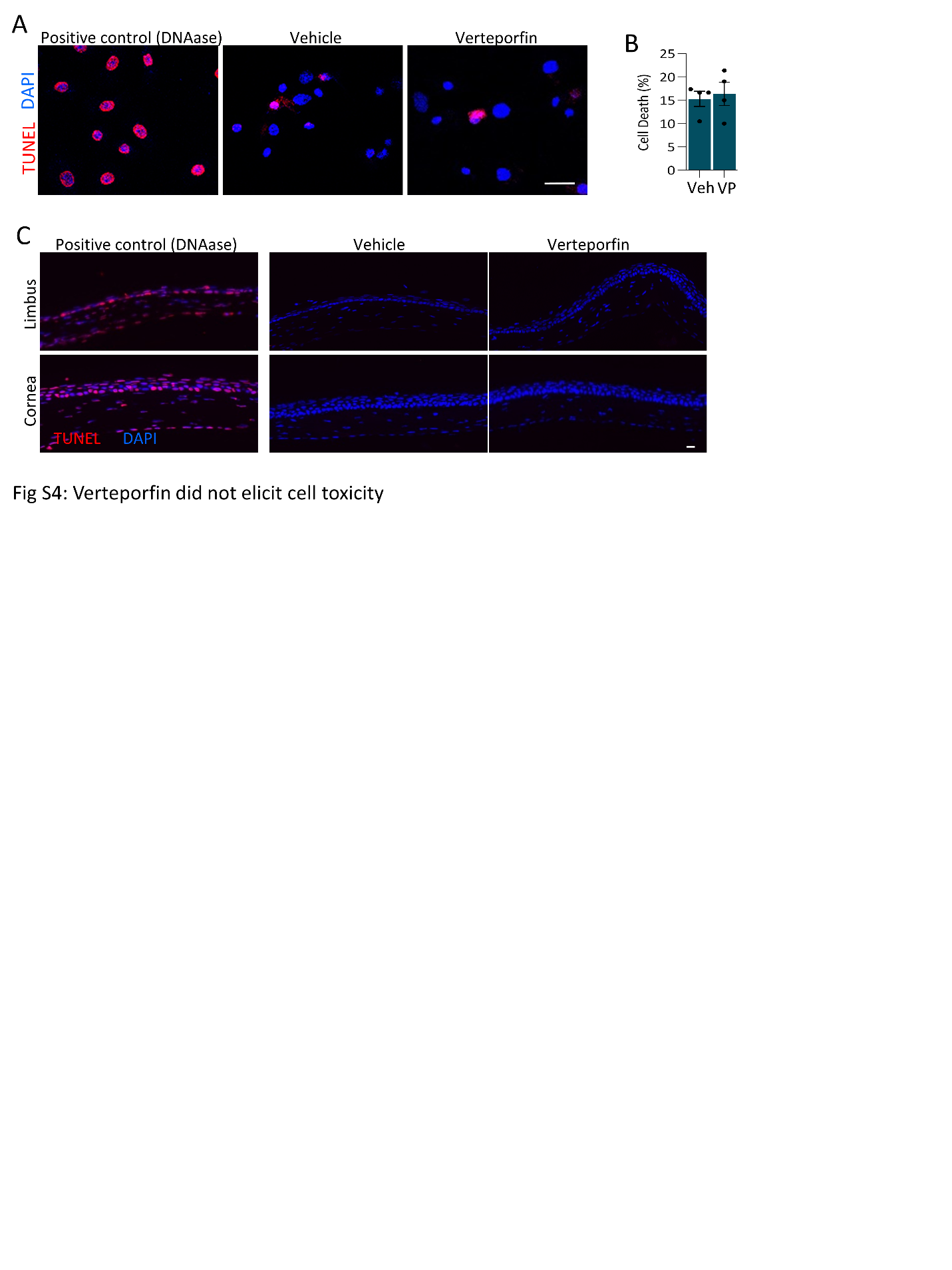
**Figure S4.** **Verteporfin did not elicit cell toxicity.** Primary LSCs were grown on silicone gels that mimic limbal rigidity in the presence of Verteporfin (VP) or Vehicle control. On the next day, cell death was assessed by TUNEL assay and cells were imaged (A) and quantified (B). (C) A daily sub-conjunctival injection (20µl) of Verteporfin (20µM) or vehicle (control) was performed for 4 days on K15-GFP transgenic mice. Tissue morphology was pictured following paraffin section preparation and apoptotic cells were detected by TUNEL assay. DNase treatment served as positive control and nuclei were detected by DAPI counterstaining. Data represented from 4 biological replicates. Scale bars are 50µm.


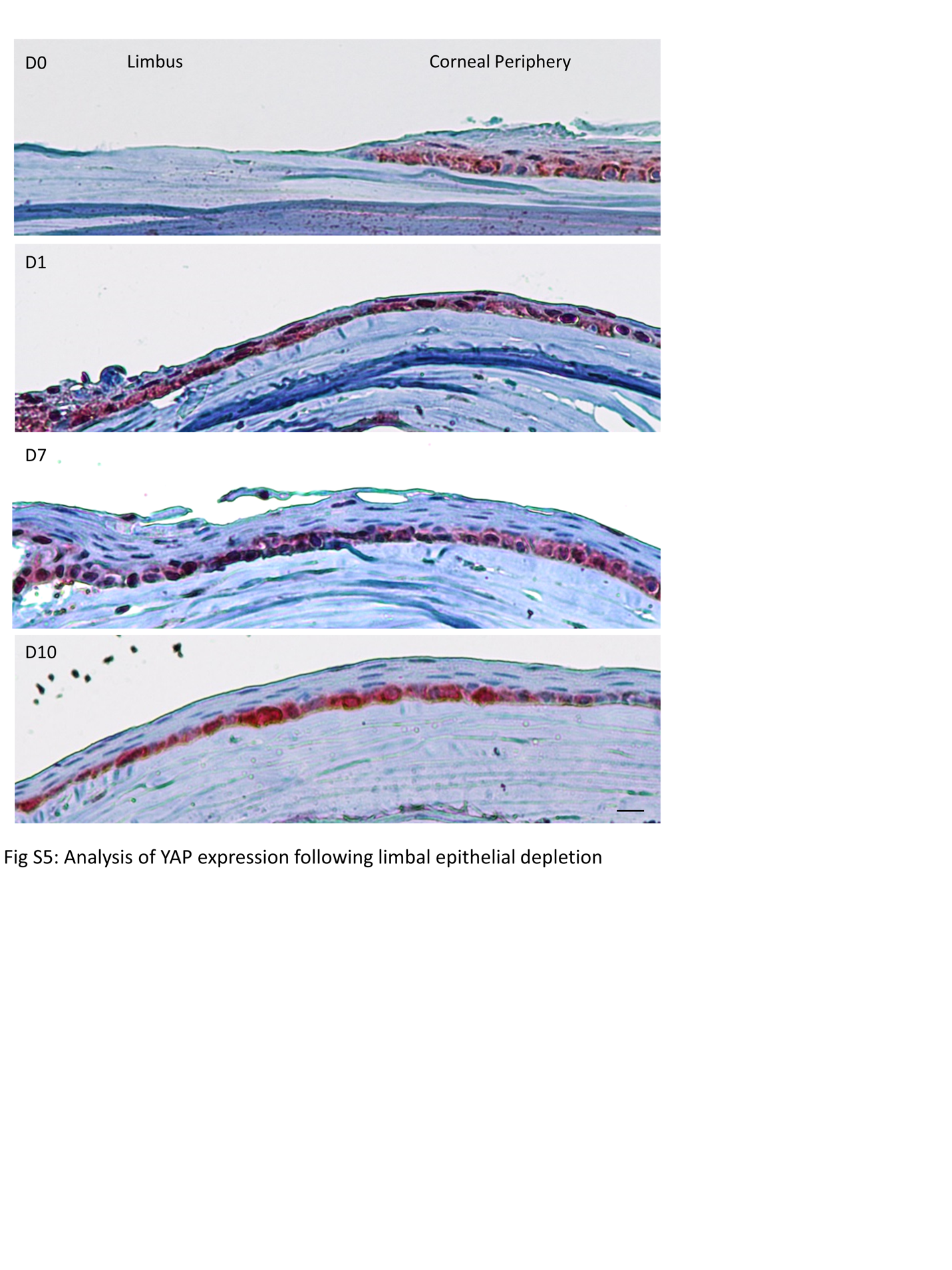


**Figure S5. Analysis of YAP expression following limbal epithelial depletion.** The limbal epithelium of adult mice was removed by Algerbrush (see Fig.3), mice were sacrificed in the indicated days post injury and immunohistochemistry was performed to detect YAP on paraffin limbus/cornea tissue sections. Immunohistochemistry data are representative from 3 biological replicates. Scale bar is 50µm.


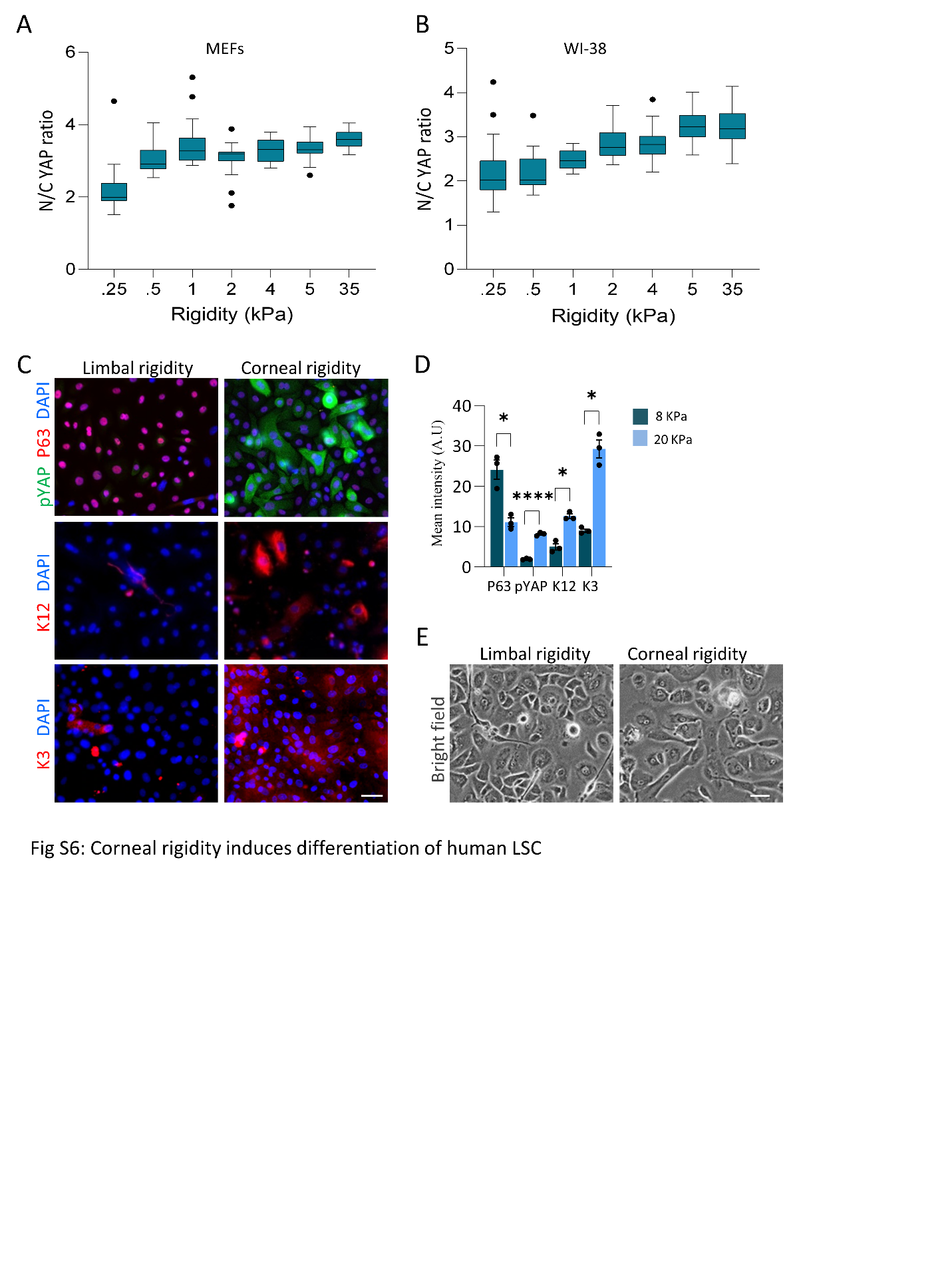
**Figure S6. Corneal rigidity induces differentiation of human LSC.** (A-B) Mean nuclear-to-cytoplasmic YAP intensity ratio of MEF (A) and WI-38 (B) cells on different rigidities for 12 hours. (C-D) Primary human limbal epithelial cells were grown for 4 days on a silicone substrate that mimics the rigidity of the limbus (8kPa) or cornea (20kPa) coated with fibronectin. Cell differentiation state was examined by immunofluorescent staining of the indicated markers (C) and mean fluorescent intensity was quantified (n=4) (D) along with morphological assessment with phase contrast images (E). Images are representative from 4 biological replicates. Statistical significance was assessed by t-test (*, *p* < .05; ****, *p* < .0001). Scale bar is 50µm.


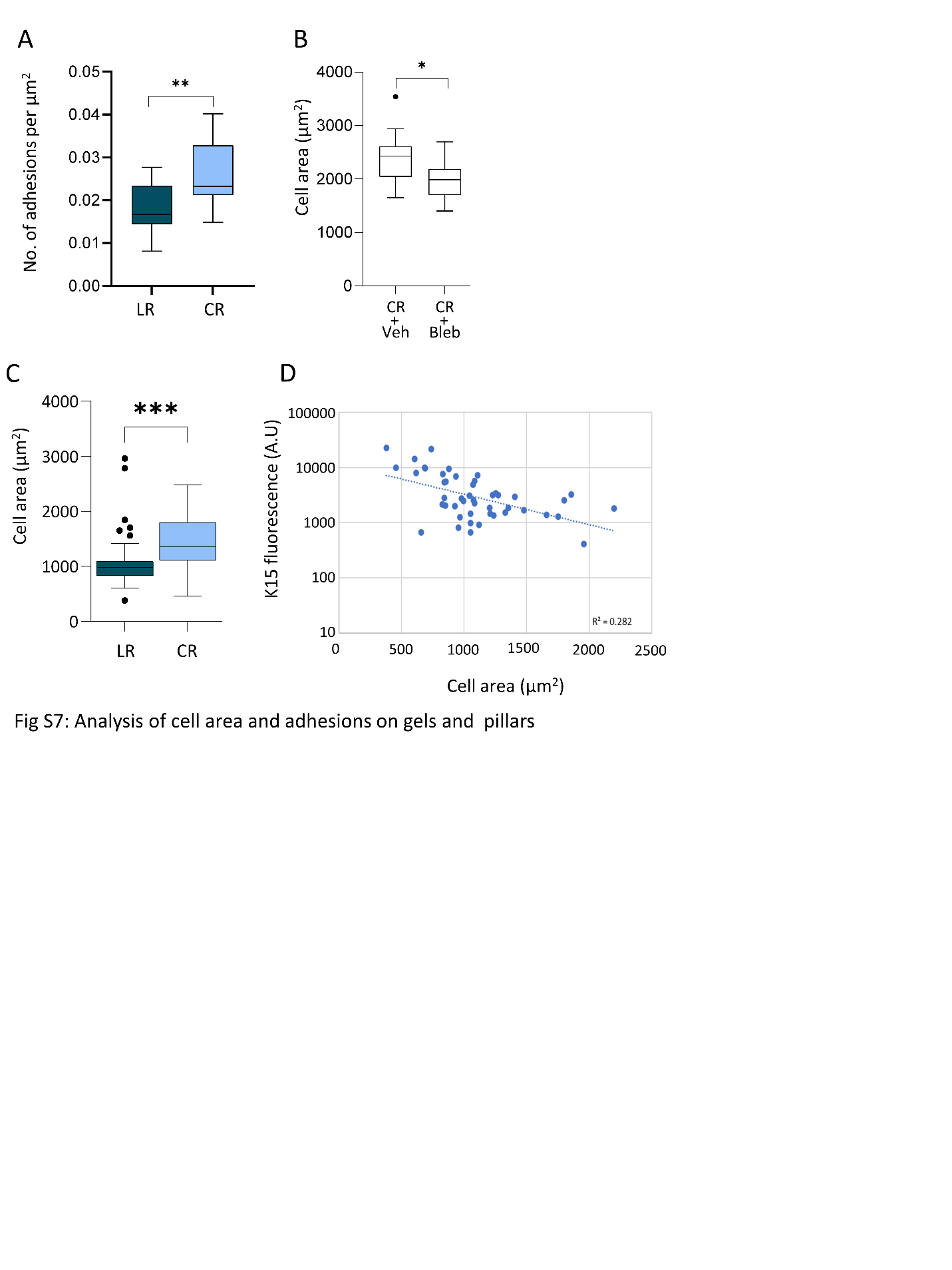


**Figure S7. Analysis of cell area and adhesions on gels and pillars.** (A) Analysis of the number of mature vinculin adhesions per µm^2^ in human limbal epithelial cells that were grown on silicone gel with limbal rigidity (LR, 8kPa) or corneal rigidity (CR, 20kPa). (B) Analysis of the areas covered by limbal cells on silicone gel with the indicated rigidity in the presence of Blebbistatin (Bleb), or vehicle (Control). (C) Areas of cells as measured using the phalloidin signal after overnight incubation on LR and CR pillars. (D) K15 immunostaining fluorescence signal as a function of cell area in cells plated overnight on the soft pillars. The number of adhesions per µm^2^ and cell area is shown by the Tukey box-and-whisker plot followed by t-test (n=3 independent experiments) (*, *p* < .05; **, *p* < .01; ***, *p* < .001).


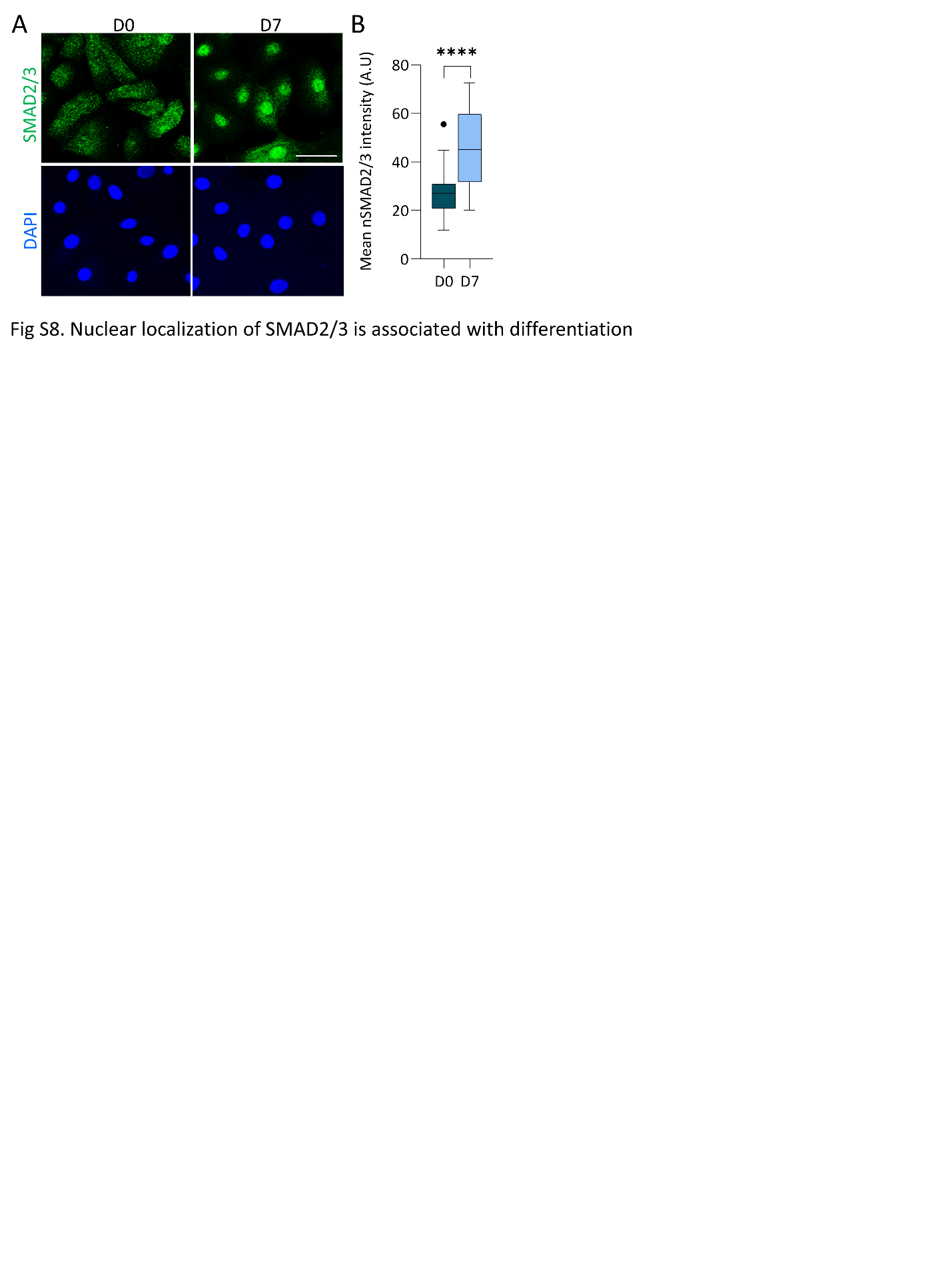


**Figure S8.** **Nuclear SMAD2/3 is associated with differentiation.** (A) Primary human LSCs were cultured on plastic culture dishes and maintained at low calcium (Day 0) or induced to differentiate in high calcium (Day7) and immunostained for SMAD2/3 (A) and mean nuclear SMAD2/3 intensity was quantified (n=3 biological replicates). (B) Mean nuclear SMAD2/3 intensity is shown by the Tukey box-and-whisker plot followed by t-test with Welch's correction (****, *p* < .0001). Data represents 3 biological replicates. Nuclei were detected by DAPI counterstaining. Scale bar is 50µm.

**Supplementary Tables**

**Supplementary Table S1.** Sequences of qRT-PCR primers.

| **Gene** | **Forward** | **Reverse** |
| --- | --- | --- |
| K15 | 5'-gacggagatcacagacctgag-3' | 5'-ctccagccgtgtctttatgtc -3' |
| P63 | 5'-gtcatttgattcgagtagagggg-3' | 5'-ctgggtggctcataaggt-3' |
| K12 | 5’-tgaatggtgaggtggtctca-3’ | 5’-tttcagaagggcaaaaagga-3’ |
| K3 | 5’-cccagcgctactccagata-3’ | 5’-ccacacctggacaatcaca-3’ |
| YAP | 5’-accctcgttttgccatgaac-3’ | 5’-tgtgctgggattgatattccg-3’ |
| HPRT1 | 5’-cctggcgtcgtgattagtgat-3’ | 5’-agacgttcagtcctgtccataa-3’ |
| ACTB | 5’-ccaccgcgagaagatga-3’ | 5’-ccagaggcgtacagggatag-3’ |
| GAPDH | 5’-gccaaggtcatccatgacaac-3’ | 5’-gtccaccaccctgttgctgta-3’ |

**Supplementary Table S2.** Source of antibodies used.

| **Antibodies** | **Source** |
| --- | --- |
| YAP (1:200) | Santa cruz biotechnology, sc-101199 (All figures except Fig. S3A, Fig. 4B) |
| YAP (1:200) | Abnova, H00010413-M01 (Fig. S3A, Fig. 4B) |
| pYAP (1:1000) | Cell signaling, 13008 |
| SMAD2/3 (1:400) | Abcam, ab202445 |
| pLATS1 (1:400) | Enco, 50-198-8119 |
| K15 (1:1000) | Santa cruz biotechnology, sc-47697 |
| K12 (1:400) | Abcam, ab185627 |
| K3 (1:400) | Abcam, ab68260 |
| PHALLOIDIN (1:400) | Thermo Fisher, A12379 |
| VINCULIN (1:300) | Thermo Fisher, 700062 |
| P63 (1:400) | Santa cruz biotechnology, sc-8431 (All figures except Fig. S1D) |
| P63 (1:100) | Abcam, ab32353 (Fig. S1D) |
| K4 (1:100) | Abcam, ab183329 |
| CD63 (1:100) | Santa cruz biotechnology, sc-5275 |
| GPHA2 (1:1000) | Santa cruz biotechnology Cat, sc-390194 |
| EDU | Invitrogen, E10187 |

**Reference:**

1. Gabay Yehezkely R, Zaffryar-Eilot S, Kaganovsky A, Fainshtain Malka N, Aviram R, Livneh I, et al. Intracellular Role for the Matrix-Modifying Enzyme Lox in Regulating Transcription Factor Subcellular Localization and Activity in Muscle Regeneration. Dev Cell [Internet]. 2020;53(4):406-417.e5. Available from: http://www.sciencedirect.com/science/article/pii/S1534580720302690

2. Amitai-Lange A, Berkowitz E, Altshuler A, Dbayat N, Nasser W, Suss-Toby E, et al. A Method for Lineage Tracing of Corneal Cells Using Multi-color Fluorescent Reporter Mice. JoVE [Internet]. 2015;(106):e53370. Available from: https://www.jove.com/video/53370

3. Wolfenson H, Meacci G, Liu S, Stachowiak MR, Iskratsch T, Ghassemi S, et al. Tropomyosin controls sarcomere-like contractions for rigidity sensing and suppressing growth on soft matrices. Nat Cell Biol. 2016 Jan;18(1):33–42.

4. Meacci G, Wolfenson H, Liu S, Stachowiak MR, Iskratsch T, Mathur A, et al. α-Actinin links extracellular matrix rigidity-sensing contractile units with periodic cell-edge retractions. Mol Biol Cell. 2016 Nov;27(22):3471–9.

5. Amitai-Lange A, Altshuler A, Bubley J, Dbayat N, Tiosano B, Shalom-Feuerstein R. Lineage tracing of stem and progenitor cells of the murine corneal epithelium. Stem Cells. 2015;33(1):230–9.

6. Ou G, Thakar D, Tung JC, Miroshnikova YA, Dufort CC, Gutierrez E, et al. Visualizing mechanical modulation of nanoscale organization of cell-matrix adhesions. Integr Biol [Internet]. 2016 Jul 11;8(7):795–804. Available from: https://doi.org/10.1039/c6ib00031b

7. Mertz AF, Che Y, Banerjee S, Goldstein JM, Rosowski KA, Revilla SF, et al. Cadherin-based intercellular adhesions organize epithelial cell-matrix traction forces. Proc Natl Acad Sci U S A. 2013 Jan;110(3):842–7.

8. Kumar A, Ouyang M, Van den Dries K, McGhee EJ, Tanaka K, Anderson MD, et al. Talin tension sensor reveals novel features of focal adhesion force transmission and mechanosensitivity. J Cell Biol. 2016 May;213(3):371–83.

9. Bergert M, Lendenmann T, Zündel M, Ehret AE, Panozzo D, Richner P, et al. Confocal reference free traction force microscopy. Nat Commun. 2016 Sep;7:12814.

10. Missirlis D, Haraszti T, Heckmann L, Spatz JP. Substrate Resistance to Traction Forces Controls Fibroblast Polarization. Biophys J. 2020 Dec;119(12):2558–72.

11. Ghassemi S, Meacci G, Liu S, Gondarenko AA, Mathur A, Roca-Cusachs P, et al. Cells test substrate rigidity by local contractions on submicrometer pillars. Proc Natl Acad Sci U S A. 2012 Apr;109(14):5328–33.

12. Feld L, Kellerman L, Mukherjee A, Livne A, Bouchbinder E, Wolfenson H. Cellular contractile forces are nonmechanosensitive. Sci Adv. 2020 Apr;6(17):eaaz6997.

13. Edelstein A, Amodaj N, Hoover K, Vale R, Stuurman N. Computer control of microscopes using µManager. Curr Protoc Mol Biol. 2010 Oct;Chapter 14:Unit14.20.
